# CREB3L4 promotes hepatocellular carcinoma progression and decreases sorafenib chemosensitivity by promoting RHEB-mTORC1 signaling pathway

**DOI:** 10.1016/j.isci.2024.108843

**Published:** 2024-01-09

**Authors:** Zhengchen Jiang, Bowen Shi, Yun Zhang, Tianming Yu, Yang Cheng, Jiankang Zhu, Guangyong Zhang, Mingwei Zhong, Sanyuan Hu, Xiaomin Ma

**Affiliations:** 1 Department of General Surgery, The First Affiliated Hospital of Shandong First Medical University & Shandong Provincial Qianfoshan Hospital, Jinan 250014, China; 2 Department of Gastric Surgery, The Cancer Hospital of the University of Chinese Academy of Sciences (Zhejiang Cancer Hospital), Institutes of Basic Medicine and Cancer, Chinese Academy of Sciences, Hangzhou 310022, China; 3 Department of General Surgery, Shandong Provincial Qianfoshan Hospital, Cheeloo College of Medicine, Shandong University, Jinan 250014, China

**Keywords:** Drugs, Molecular biology, Cancer

## Abstract

This study was designed to explore the roles of CREB3L4 in the pathogenesis and drug resistance of hepatocellular carcinoma (HCC). The proliferation of HCC lines was determined in the presence of CREB3L4 over-expression and silencing. Chromatin immunoprecipitation (ChIP) assay and dual-luciferase reporter assay were performed to screen the potential target of CREB3L4 on mTORC1. Xenografted tumor model was established to define the regulatory effects of CREB3L4 in the tumorigenesis. Then we evaluated the roles of CREB3L4 in chemosensitivity to sorafenib treatment. CREB3L4 significantly induced the HCC cell proliferation by modulating the activation of mTROC1-S6K1 signaling pathway via binding with RHEB promoter. Moreover, CREB3L4 dramatically inhibited the chemosensitivity to sorafenib treatment via up-regulating RHEB-mTORC1 signaling. CREB3L4 promoted HCC progression and decreased its chemosensitivity to sorafenib through up-regulating RHEB-mTORC1 signaling pathway, indicating a potential treatment strategy for HCC through targeting CREB3L4.

## Highlights

• CREB3L4 is associated with poor prognosis in HCC
• CREB3L4 promotes proliferation of HCC through RHEB/mTORC1 axis
• CREB3L4 reduces chemosensitivity of sorafenib in HCC through RHEB/mTORC1 axis

## Graphical abstract

**Figure:**
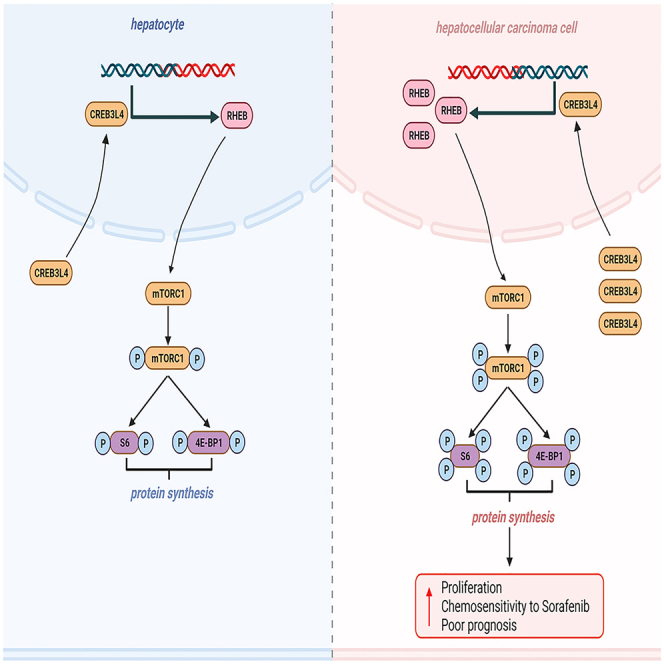

## Introduction

The main pathways involved in the pathogenesis of hepatocellular carcinoma (HCC) include Hedgehog, vascular endothelial growth factor (VEGF), hepatocyte growth factor/c-MET, WNT/b-catenin, mitogen-activated protein kinase (MAPK)/ERK, as well as PI3K/AKT/mTOR.^1^^,^^2^ Among these pathways, particular interests have been paid to the PI3K/AKT/mTOR pathway because there is constitutive activation in many HCC patients with aggressive tumor progression and reduced survival.^3^ Moreover, it may decrease the susceptibility of HCC cells to the sorafenib-based target therapy.^4^ Up to now, several upstream elements have been reported to regulate the mTORC1 pathway including RHEB and TSC1/TSC2 complex. TSC1/TSC2 complex, as the upstream of RHEB, could inhibit the RHEB-mediated phosphorylation of mTORC1,^5^ which was crucial for HCC pathogenesis and progression, as well as drug resistance.

Cyclic AMP response element binding (CREB) protein, first identified in 1987 during the investigation for nuclear proteins binding to a stretch of DNA, is over-expressed in many cancers by acting as a transcriptional factor entailing the protein-DNA and protein-protein interactions in a certain signaling pathways (e.g., Ras/MAPK, PKA, and PI3K/AKT).^6^ Recently, CREB3 family comprised five members in mammals including CREB3, CREB3L1, CREB3L2, CREB3L3, and CREB3L4 have been reported to involve in an overall range of functions such as metabolism, survival, differentiation and tumorigenesis.^7^ Among these members, CREB3L4, also known as CREB4, was first described in 2002 by two independent research groups.^8^^,^^9^ Its main function is related to the proliferation of prostate cancers promoted by androgen receptor and IRE1-ɑ. In addition, downstream target genes of CREB3L4 were closely associated with the cellular proliferation, which then resulted in the tumorigenesis.^10^ Aberrant CREB3L4 expression had been observed in prostate cancer and gastric cancer. However, rare studies have focused on the mechanism of how CREB3L4 involves in the HCC except one study, in which Wang et al. indicated that CREB3L4 promoted the angiogenesis of tumor progression in gastric cancer via regulating vascular endothelial growth factor A (VEGFA).^11^

Sorafenib is one of the main targeted drugs for the systemic treatment of advanced HCC. However, only one-third of patients respond to sorafenib, with the majority showing recurrence within 6 months.^12^ To date, HCC sensitivity to sorafenib involves hypoxia-related pathway, energy metabolism, natural compounds and nano-particles, autophagy as well as microRNAs.^13^^,^^14^^,^^15^^,^^16^ Nevertheless, no studies have focused on the effects of CREB3L4 on the sorafenib sensitivity in HCC. This study was designed to investigate the roles of CREB3L4 expression in the pathogenesis and drug resistance of HCC cells, and the function of the mTORC1 pathway in this process.

## Results

### CREB3L4 up-regulation was positively correlated with a poor prognosis among HCC patients

**Figure 1:**
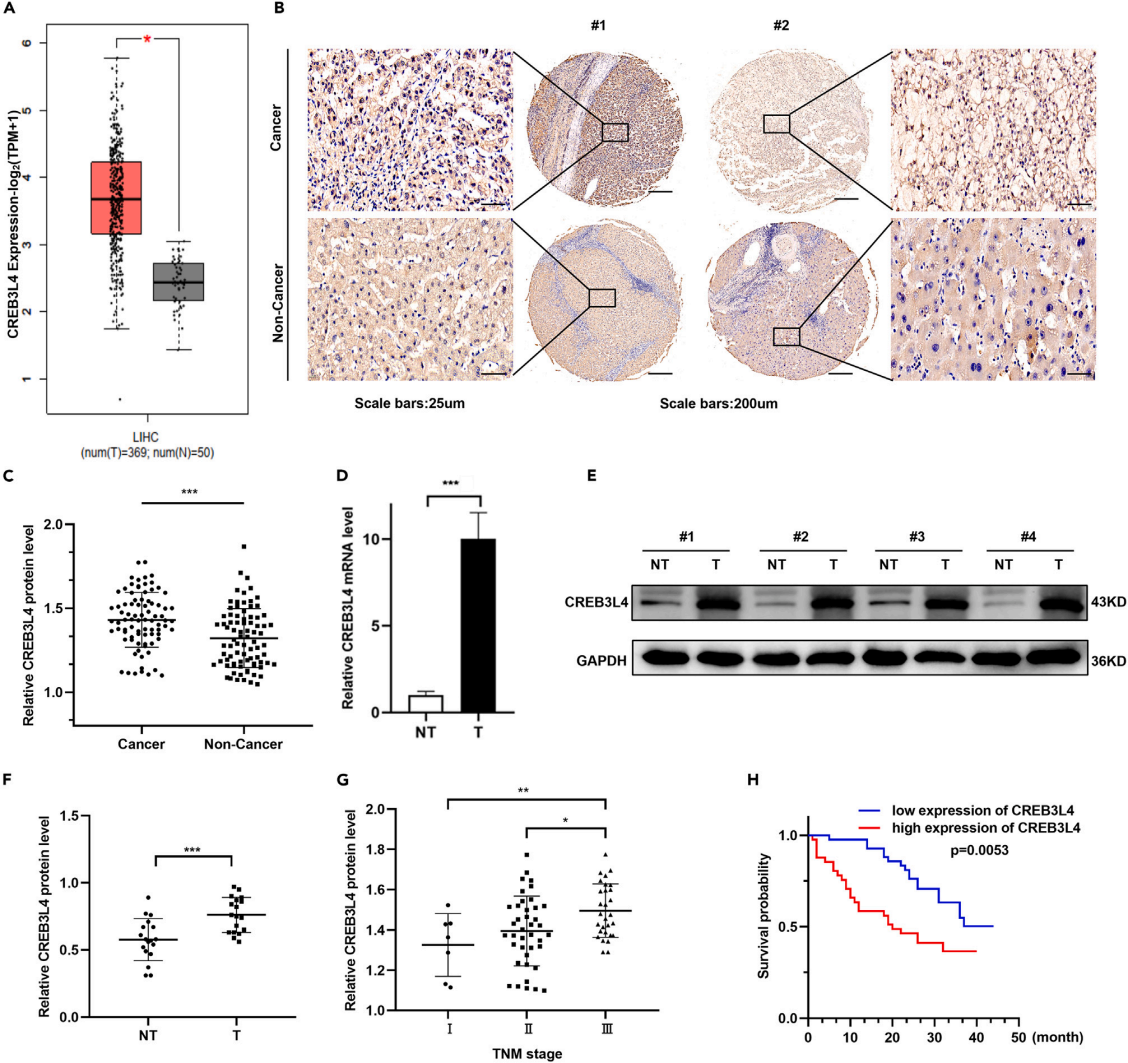
Upregulation of CREB3L4 was correlated with poor prognosis in HCC patients (A) Boxplots of *CREB3L4* mRNA expression in tumor tissues and the non-tumor tissues of HCC patients from TCGA database. (B) Eighty-three pairs of HCC tissues and matched distal non-cancerous liver tissues were collected for IHC staining to investigate the expression level of CREB3L4. IHC staining showed CREB3L4 expression was significantly increased in HCC tissues compared with the corresponding normal tissues. The representative images of CREB3L4 expression in the clinical specimens were presented. (C) The IHC data of CREB3L4 expression in cancerous and non-cancerous liver tissues was further quantitatively analyzed by ImageJ software. (D) qRT-PCR analysis was conducted to measure the relative expression level of the CREB3L4 mRNA in tumor tissues (T) and the normal tissues (NT) of 20 pairs of HCC patients. (E and F) Western blot analysis was conducted to measure the relative expression level of the CREB3L4 protein in tumor tissues (T) and the normal tissues (NT) of 20 pairs of HCC patients. Western blot data of CREB3L4 expression was quantitatively analyzed by ImageJ software. (G) Representative images showed the IHC staining of CREB3L4 in normal and HCC tissues at different stages (I = 7, II = 47, and III = 29). (H) The overall survival of HCC patients with low (n = 41) and high CREB3L4 expression (n = 42) was analyzed using log rank test. ∗p < 0.05, ∗∗p < 0.01, ∗∗∗p < 0.001 for statistical analysis of the indicated groups.

The Cancer Genome Atlas (TCGA) database showed significant up-regulation of *CREB3L4* mRNA in HCC tissues compared with non-cancerous liver tissues (Figure 1A). Western blot analysis and IHC results showed that the CREB3L4 protein was also significantly up-regulated in HCC tissues compared with the matched normal tissues (Figures 1B–1F). In addition, IHC results further confirmed a positive association between CREB3L4 expression and the TNM stages of HCC (Figure 1G). Meanwhile, HCC patients with a high CREB3L4 expression showed a shorter overall survival than the counterparts with low CREB3L4 expression (Figure 1H).

### CREB3L4 promoted cellular proliferation and colony formation of HUH7 cells and Hep3B cells

**Figure 2:**
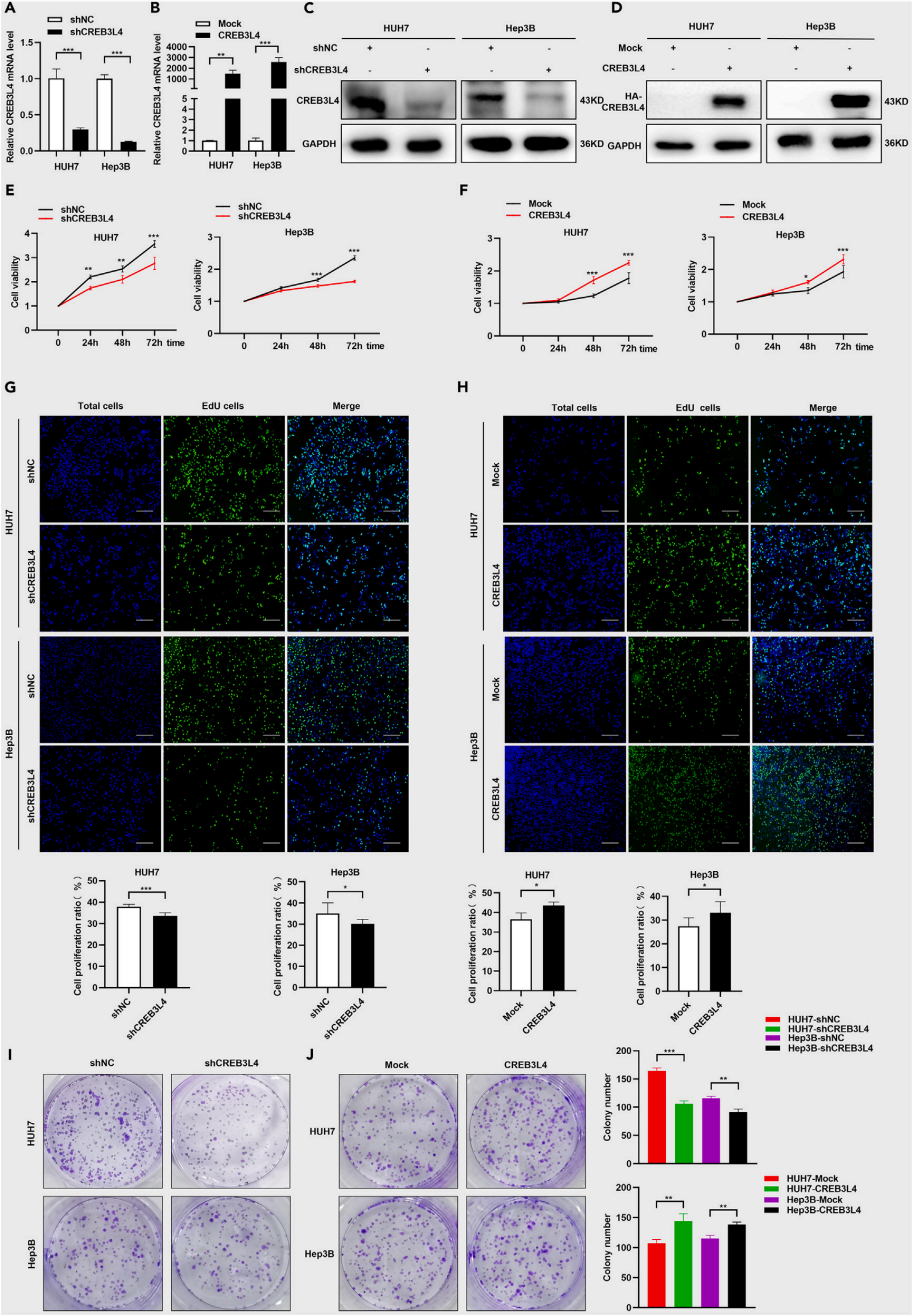
CREB3L4 significantly promoted the proliferation and colony formation in HCC cells Gain-of-function and loss-of-function cell models were established based on transfection with shCREB3L4 and HA-CREB3L4. (A–D) The qRT-PCR and western blot assays were performed to detect the effective knockdown and over-expression of CREB3L4 at 48 h after the transfection. (E and F) CCK-8 assay was performed to detect the cell proliferation status of these shCREB3L4-transfected HCC cells or HA-CREB3L4 transfected cells at 0, 24 h, 48 h, and 72 h. (G and H) Cell proliferation status was detected by EdU assays after knockdown or over-expression of CREB3L4. The original magnification was 20×. (I and J) Colony formation assay was performed to detect the colony formation ability of these shCREB3L4-transfected HCC cells and HA-CREB3L4 transfected cells. ∗p < 0.05, ∗∗p < 0.01, ∗∗∗p < 0.001 for statistical analysis of the indicated groups.

To investigate the roles of CREB3L4 in HCC progression, we determined its basal expression in HUH7 and Hep3B cells (Figures S2A and S2B). Loss-of-function and gain-of-function cellular models of CREB3L4 were established based on HCC cells using shCREB3L4 plasmid and CREB3L4 plasmid, respectively. The construction of loss-of-function model (Figures 2A and 2B) and gain-of-function (Figures 2C and 2D) were verified by real-time PCR and western blot analysis. CCK-8 assay (Figure 2E), EdU staining (Figure 2G), and colony formation assay (Figure 2I) showed dramatical decrease in the proliferation of HCC cells in CREB3L4 loss-of-function cellular model. The proliferation of HUH7 and Hep3B cells was significantly increased in the presence of CREB3L4 over-expression in HCC cells (Figures 2F, 2H, and 2J). Both the loss-of-function and gain-of-function of CREB3L4 cellular models demonstrated that CREB3L4 promoted the proliferation of HUH7 and Hep3B cells.

### CREB3L4 promoted the HCC proliferation by up-regulating mTORC1 signaling pathway in HCC cells

**Figure 3:**
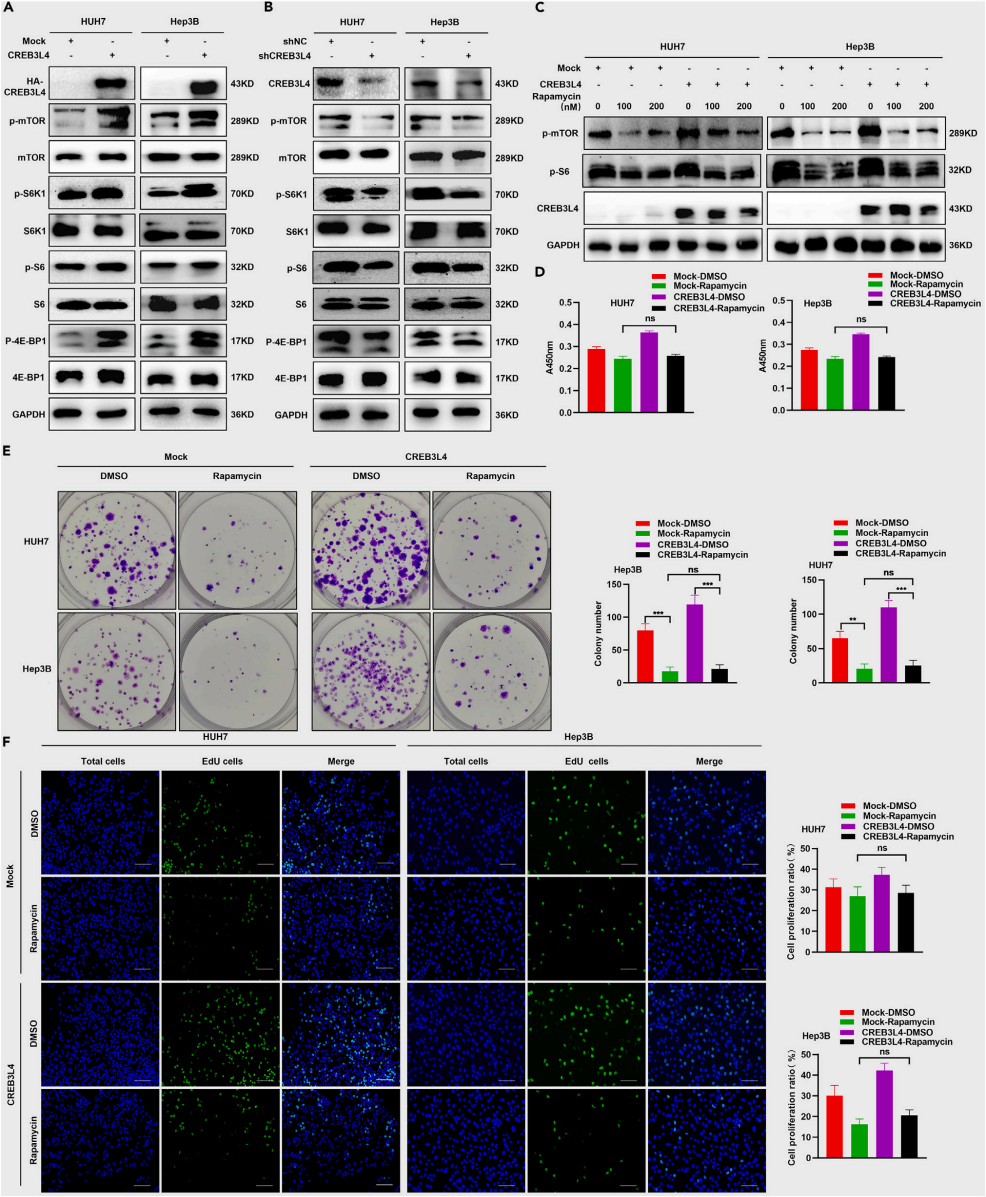
CREB3L4 up-regulated the mTORC1 signaling pathway (A and B) HUH7 and Hep3B cells were transfected with CREB3L4 or shCREB3L4 plasmid. Approximately 48 h after transfection, western blot was utilized to measure the expression of mTOR signaling associated proteins including *p*-mTOR, mTOR, p-S6K1, S6K1, p-S6, S6, p-4E-BP1, and 4E-BP1. (C) HUH7 cells were transfected with CREB3L4 and rapamycin (0, 100 nM, and 200 nM) serving as an inhibitor of mTOR signaling. About 48 h after transfection, western blot assay was performed to detect the levels of *p*-mTOR and CREB3L4. (D) CCK-8 assay was performed to detect the cell viabilities of these CREB3L4-transfected HCC cells at 48 h after transfection. (E) Colony formation assay was performed to detect proliferation of these CREB3L4-transfected HCC cells. (F) Cell proliferation status was detected by EdU assay. The original magnification was 20×. Presented figures were representative data from three independent experiments. ∗∗∗p < 0.001 for statistical analysis of the indicated groups.

To investigate the signaling pathway participated in the cellular proliferation induced by CREB3L4, we determined the protein expression in the classical signaling pathways implicated with cellular proliferation (i.e., AKT, MAPK and mTORC1). Our data showed that CREB3L4 over-expression did not activate the AKT and MAPK signaling pathway (data not shown). Whereas, its over-expression could significantly up-regulate the phosphorylation of mTROC1 and its downstream elements such as S6K1, S6 and 4E-BP1 (Figure 3A). Consistently, CREB3L4 knockdown triggered significant inhibition in mTROC1 signaling pathway and the downstream elements (Figure 3B). To investigate how CREB3L4 modulated the activation of mTORC1 signaling pathway, HUH7 and Hep3B cells were incubated with the rapamycin serving as a specific inhibitor for blocking the mTORC1 signaling pathway. Up-regulation of mTORC1 signaling pathway mediated by CREB3L4 was significantly inhibited in the presence of rapamycin (Figure 3C). In addition, rapamycin pre-treatment could reverse the proliferation of HCC cells promoted by CREB3L4, which was featured by decreased cellular viability (Figure 3D) and colony formation (Figure 3E). IHC indicated no significant differences between the EdU-stained cell number between the mock group treated with rapamycin and the CREB3L4 group treated with rapamycin (Figure 3F). Combination index values confirmed that CREB3L4 knockdown showed synergistic effects on rapamycin (Figure S3A). All these confirmed that CREB3L4 promoted the proliferation of HCC cells via up-regulating the mTORC1 signaling pathway.

### CREB3L4 up-regulated mTORC1 signaling pathway via enhancing the transcriptional activity of RHEB

**Figure 4:**
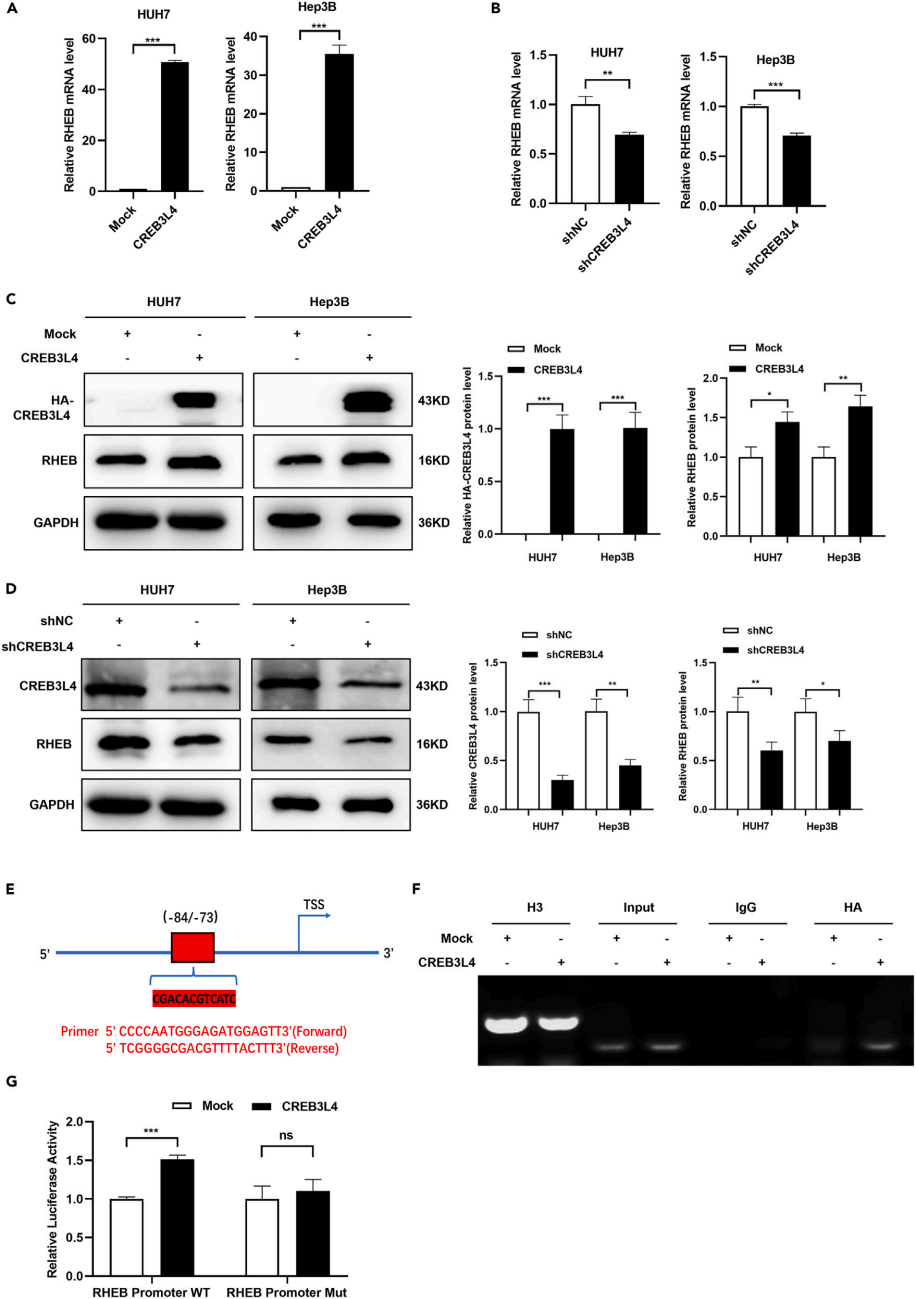
CREB3L4 upregulated mTORC1 signaling pathway via increasing the transcriptional activity of RHEB (A–D) HUH7 and Hep3B cells were transfected with CREB3L4 and shCREB3L4 for 48 h, and vector and shNC plasmids served as mock control, respectively. The expression of RHEB mRNA and protein was detected by qRT-PCR and western blot analysis. Western blot data of CREB3L4 expression was quantitatively analyzed by ImageJ software. (E) The binding motif of CREB3L4 at the promoter of RHEB and the primer sequences for ChIP assay. (F) Chromatin immunoprecipitation was performed using anti-HA or control IgG antibodies. The binding status between CREB3L4 and *RHEB* promoter was detected by PCR. (G) HUH7 cells were co-transfected with CREB3L4 expression plasmid or mock control, RHEB wild-type (WT) or mutation (Mut) promoter-luciferase reporter plasmid and pRL-TK plasmid. The cells were harvested to luciferase activity analysis using a dual-luciferase reporter assay 48 h after the transfection. ∗∗p < 0.01, ∗∗∗p < 0.001 for statistical analysis of the indicated groups.

It has been well known that mTORC1 is activated by small GTPase RHEB as the active GTP-bound form of RHEB could interact with mTORC1 and allosterically switch on mTOR kinase activity (22). Besides, based on the TCGA database, we found a positive correlation between RHEB and CREB3L4 (data not shown). Therefore, we speculated that *RHEB* gene was the target of CREB3L4 in HCC cell lines. To our expectation, RHEB mRNA and protein expression showed significant up-regulation in the presence of CREB3L4 over-expression, while CREB3L4 knockdown significantly down-regulated the expression of RHEB mRNA and protein (Figures 4A–4D). As CREB3L4 is a transcriptional regulator, we then investigated the regulation of CREB3L4 on the transcriptional activity of RHEB. The binding motif of CREB3L4 at the promoter of *RHEB* and the primer sequences for chromatin immunoprecipitation (ChIP) assay were showed in Figure 4E. ChIP assay indicated that CREB3L4 could bind with the promoter of *RHEB* (Figure 4F). Besides, dual-luciferase assay showed that CREB3L4 could significantly up-regulate the transcriptional activity of *RHEB* in HCC cells (Figure 4G). Finally, RHEB knockdown reversed the promoting effects of CREB3L4 on the proliferation of cancer cells (Figures S4A and S4B). All these indicated that CREB3L4 promoted the activation of mTORC1 signaling pathway through up-regulating the transcriptional activity of *RHEB*.

### CREB3L4 decreased the chemosensitivity of HUH7 cells and Hep3B cells to sorafenib by up-regulating RHEB-mTORC1 axis

**Figure 5:**
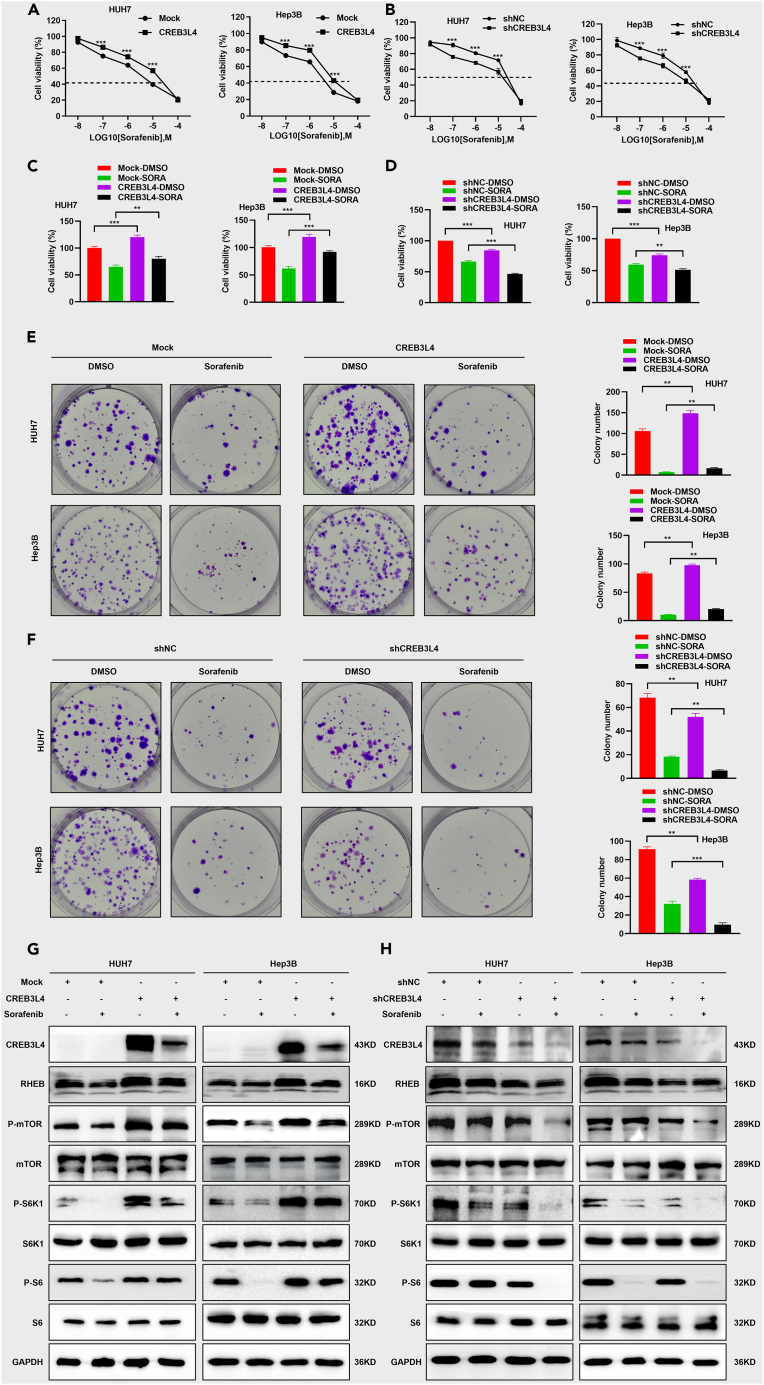
CREB3L4 significantly decreased the chemosensitivity of HCC cells to sorafenib by up-regulating RHEB-mTORC1 axis HUH7 and Hep3B cells were transfected with shCREB3L4 and HA-CREB3L4. (A and B) Cells were treated with different dosage (0, 0.01 μM, 0.1 μM, 1 μM, 10 μM, and 100 μM) of sorafenib for 48 h, then CCK-8 assay was performed to detect cells viabilities. (C and D) Cells were treated with sorafenib (10 μM) for 48 h, then CCK-8 assay was performed to detect cells viability. (E and F) Colony formation assay was performed to detect the proliferation of the cells treated with sorafenib (10 μM). (G and H) HUH7 and Hep3B cells were transfected with CREB3L4 or shCREB3L4. Approximately 48 h after transfection, the expression levels of mTOR signaling associated proteins including RHEB, *p*-mTOR, mTOR, p-S6K1, S6K1, p-S6, S6, p-4E-BP1, and 4E-BP1 were detected. ∗∗p < 0.01, ∗∗∗p < 0.001 for statistical analysis of the indicated groups.

CREB3L4 over-expression dramatically reduced the HCC cell chemosensitivity to sorafenib, while CREB3L4 silencing significantly increased the chemosensitivity of HCC cells to sorafenib (Figures 5A–5D). Similarly, the sorafenib-treated HCC cells with overexpression of CREB3L4 showed significant increase in cell proliferation and colony formation compared with the mock-Sorafenib or shNC-Sorafenib group. HCC cells with CREB3L4 silencing showed significant decrease in cell proliferation and colony formation compared with the mock-Sorafenib or shNC-Sorafenib group (Figures 5E and 5F). Combination index values confirmed that CREB3L4 knockdown showed synergistic effects on sorafenib (Figure S5A). To investigate the potential mechanisms involved in the decreased chemosensitivity of HCC cells to sorafenib, we then determined the expression of proteins in the RHEB-mTORC1 signaling axis. Western blot showed remarkable down-regulation in the expression of RHEB, phosphorylation of mTORC1, S6K1 and S6 in sorafenib-treated HCC cells with CREB3L4 knockdown (Figures 5G and 5H). Thus, CREB3L4 decreased HCC chemosensitivity to sorafenib treatment through up-regulating RHEB-mTORC1 axis.

### CREB3L4 promoted tumorigenesis and decreased chemosensitivity of HCC to sorafenib in xenografted tumor models

**Figure 6:**
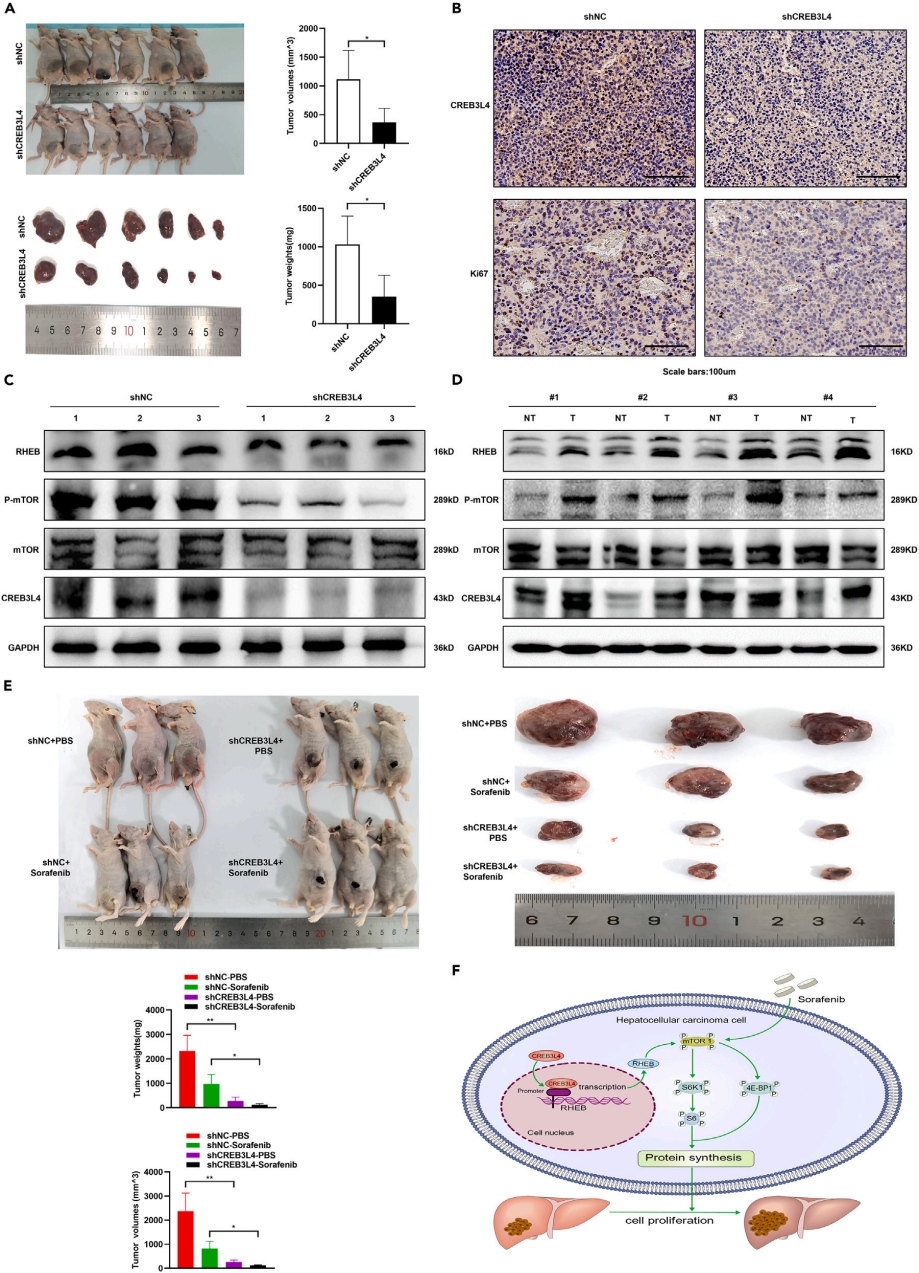
CREB3L4 promoted tumorigenesis and decreased chemosensitivity of HCC cells to sorafenib treatment in HCC xenografted tumor models (A) HUH7 cells (8×10^6^) infected with LV-shCREB3L4 or LV-shNC were subcutaneously injected to the left flanks of the nude mice (n = 6 for each group). Images presented were the tumor-bearing BALB/c nude mice and their dissected tumors. Tumor volumes and tumor weights were measured and statistically analyzed. (B) The relative expression of CREB3L4 and Ki67 was detected by IHC. (C) The relative expression level of CREB3L4, RHEB, *p*-mTOR and mTOR expression was detected by western blot. (D) The relative expression level of the CREB3L4, RHEB, *p*-mTOR and mTOR in tumor tissues (T) and the normal tissues (NT) of HCC patients was detected by western blot. (E) HUH7 cells (8 × 10^6^) infected with LV-shCREB3L4 or LV-shNC were subcutaneously injected to the left flanks of the nude mice (n = 6 for each group). When visible tumor appeared, animals were randomly divided into two groups including sorafenib treatment group and PBS treatment group. Two sorafenib treatment groups (n = 3 for each group) were injected with sorafenib (5 mg/kg) for every 3 days and two PBS treatment groups were injected with same volume PBS. Representative images were the tumor-bearing BALB/c nude mice and their dissected tumors. Tumor volumes and tumor weights of four groups were measured and statistically analyzed. (F) Working model showing the roles and mechanisms of CREB3L4 in HCC. CREB3L4 promoted HCC progression and decreased chemosensitivity through up-regulating RHEB-mTORC1 signaling pathway. ∗p < 0.05, ∗∗p < 0.01 for statistical analysis of the indicated groups.

To further verify the roles of CREB3L4 in progression and chemosensitivity of HCC under *in vivo* conditions, we constructed HCC xenografted tumor models based on LV-shCREB3L4 and LV-shNC HCC cell lines. HCC xenografted models established based on LV-shCREB3L4 transfected cells showed significant decrease in the tumor volume and tumor weight compared with those with models established based on LV-shNC transfected cells (Figure 6A). Knockdown of CREB3L4 in the xenografts models based on injection of LV-shCREB3L4 transfected cells could significantly inhibit the proliferation of HCC cells (Figure 6B). RHEB-mTORC1 axis was significantly down-regulated in LV-shCREB3L4 xenografted models compared with that of LV-shNC xenografted models (Figure 6C). Besides, compared with the non-cancerous liver tissues, the expression of CREB3L4 and RHEB/mTORC1 was significantly up-regulated in human HCC specimens (Figure 6D). All these data showed that CREB3L4 may promote HCC tumorigenesis through up-regulating RHEB-mTORC1 axis.

To determine the effects of CREB3L4 on the HCC chemosensitivity to sorafenib *in vivo*, the xenografted LV-shCREB3L4 or LV-shNC tumor models were treated with sorafenib or PBS, respectively. Our data showed that CREB3L4 could significantly decrease the chemosensitivity of HCC to sorafenib, which was featured by significant decrease in the tumor volume and tumor weight in the shCREB3L4-Sorafenib group compared with the other three groups, especially the shNC-Sorafenib group (all p < 0.05, Figure 6E).

## Discussion

Aberrant CREB3L4 expression is associated with the pathogenesis of HCC, prostate cancer and gastric cancer, but its targets in the pathogenesis of these cancers are still not well defined. Up to now, only one study indicated that CREB3L4 involved in the tumor progression in gastric cancer via modulating VEGFA expression.^11^ Our study, for the first time, showed that CREB3L4 could bind with the promoter of *RHEB* in the nucleus of HCC cells, which then induced the transcriptional activity of RHEB and subsequent activation of mTORC1-S6K1 axis. This process was proved to be crucial for HCC tumorigenesis and decreased chemosensitivity to sorafenib.

CREB3L4, as a member of the basic leucine zipper (bZIP) transcription factor, is reported to involve in many physiological and pathological processes by regulating the transcriptional activity of target genes.^17^ It has been recognized as a marker for predicting the prognosis of human cancers because of its high expression in prostate cancer, gastric cancer, and HCC, triggering excessive proliferation of cancer cells.^18^ In prostate cancer, CREB3L4 is considered to facilitate the prostatic cancer cell proliferation via interacting with the androgen receptor.^19^ Besides, high CREB3L4 is overexpressed in prostate cancer and gastric cancer patients with a poor overall survival.^8^ Consistent with these literatures, our data showed that over-expression of CREB3L4 enhanced the cell growth of HUH7 and Hep3B cells, while its knockdown impeded cell proliferation and attenuated cell motility. Our data showed that CREB3L4 expression was significantly up-regulated in HCC tissues compared with matched non-cancerous liver tissues. In addition, exogenous CREB3L4 inhibition *in vivo* significantly inhibited the proliferation of HCC cells, indicating that CREB3L4 could function as a tumor promoter in HCC.

The exact mechanisms of how CREB3L4 involves in the pathogenesis of malignancies are still not well defined, due to a lack of outstanding researches. Qi et al. reported that the CREB highly expressed in prostate tumors associated with the androgen receptor signaling pathway.^8^ For the mechanism, the CREB3L4 could bind to the promoter of *VEGFA* gene.^8^^,^^11^ Indeed, VEGF is one of the main pathways involved in the pathogenesis of HCC as it triggers the angiogenesis and vascular permeability of cancer cells, as well as cellular proliferation.^20^ In our study, we focused on the potential association between CREB3L4 and PI3K/AKT/mTOR pathway in the HCC pathogenesis as it is constitutively activated in a significant proportion of HCC patients, together with significant upregulation in the HCC tissues compared with the adjacent tissues according to our clinical experience and bioinformatic analysis. Therefore, we established over-expression and silencing of CREB3L4 cellular and animal models, and then investigated the potential regulation on the mTORC1 signaling pathway.

Cytoplasmic intermediates (e.g., AKT/MTOR and RAS/MAPK) and differentiation cascades (e.g., Wnt/beta catenin and Notch) have been reported to be deregulated in human HCC.^21^ The mTORC1 signaling pathway is essential for the HCC progression as its over-activation can trigger excessive proliferation and metastasis.^22^ The phosphorylation of the key substrate molecules of mTORC1 (i.e., S6K1 and the eukaryotic translation initiation protein 4E-BP1) is crucial for the protein translation for cellular proliferation, metabolism, and metastasis of HCC cancer cells.^23^^,^^24^ Therefore, inhibiting the activation of mTORC1 signaling pathway in HCC cells can effectively inhibit the tumorigenesis of HCC. It has been well acknowledged that PI3K/Akt signaling pathway is a crucial upstream signaling pathway for mTOR1.^25^ AKT signaling pathway-mediated protein synthesis and cellular proliferation could reach the GTPase-activating protein (GAP) TSC1/2.^26^ The phosphorylation of TSC2 would trigger the deactivation of TSC complex, which was designated as a GAP complex consisting of TRE2-BUB2-CDC16 domain family member 7 (TBC1D7) and TSC1/2. In contrast, the inactivation of TSC2 would enhance the activation of RHEB that directly switched on the assembly of mTORC1, which then triggered the protein synthesis that may involve in the pathogenesis and progression of HCC. Our data showed that CREB3L4 could enhance the transcriptional activity of RHEB by targeting the promoter of *RHEB* gene. Upon the activation of RHEB, the mTORC1 signaling pathway was significantly up-regulated, yielding more synthesized protein for the proliferation of HCC cells (Figure 6F). Previous study showed that CREB3L4 promoted the migration and invasion of gastric cancer and prostate cancer.^27^ However, in our preliminary experiments, the effects of CREB3L4 on the migration and invasion of HCC were not stable. In the future, more attempts should be given to it.

As a multi-kinase inhibitor that induces apoptosis, attenuates angiogenesis and inhibits tumor cell proliferation,^28^ sorafenib has been commonly used as the first-line chemotherapy drug for advanced HCC treatment.^29^ However, the effect of most patients with sorafenib treatment is unsatisfactory due to chemoresistance.^30^^,^^31^ One of the major mechanisms is the over-activation of PI3K/AKT/mTOR signaling pathway.^14^ Previous studies showed that sorafenib combined with mTORC1 inhibitor therapy can significantly enhance chemosensitivity in HCC patients.^32^^,^^33^ This implied that mTORC1 pathway played a pivotal role in the chemoresistance of HCC cells to sorafenib. In this study, CREB3L4 could significantly promote the activation of mTORC1 signaling pathway. Thus, we further investigated the regulatory effects of CREB3L4 on the chemosensitivity of HCC cells treated with sorafenib. Our study showed that CREB3L4 can significantly decrease the chemosensitivity of HCC cells to sorafenib. This indicated that the combination of CREB3L4 and sorafenib may benefit to the HCC patients, as CREB3L4 may yield to increased chemosensitivity of HCC cells to the sorafenib. In the future, more studies are required to identify the efficiency of CREB3L4 as a new therapeutic target of HCC, together with the appropriate application of anti-cancer therapy for the patients with high CREB3L4 expression.

### Conclusions

CREB3L4 was significantly up-regulated in HCC tissues and cell lines. High CREB3L4 expression was correlated with poor prognosis in HCC. CREB3L4 promoted tumorigenesis of HCC by up-regulating RHEB-mTORC1 axis. In addition, CREB3L4 significantly decreased the HCC chemosensitivity to sorafenib via activating RHEB-mTORC1 axis under *in vitro* and *in vivo* conditions. Therefore, CREB3L4 may serve as a new therapeutic target of HCC, which provides a novel treatment strategy for HCC by inhibiting CREB3L4/RHEB/mTORC1 axis.

### Limitations of the study

For the limitation of the study, we did not utilize the inhibitor of mTORC1 signaling pathway in the animal models, with which to investigate whether CREB3L4 could facilitate malignancy through activating mTORC1 pathway, or reduce the sensitivity of cancer cells to sorafenib. In addition, there is a lack of molecular validation on the roles of mTORC1 signaling pathway in HCC patients with or without sorafenib resistance. In the future, we will focus on this.

## STAR★Methods

### Key resources table

**Table:** 

REAGENT or RESOURCE	SOURCE	IDENTIFIER
**Antibodies**		

Phospho-mTOR (Ser2448)	Cell Signaling Technology	Cat#5536S; RRID: AB_2792875
mTOR	Cell Signaling Technology	Cat#2983S; RRID: AB_2105650
Phospho-Drosophila p70 S6 Kinase (Thr398)	Cell Signaling Technology	Cat#9209S; RRID: AB_2269804
p70 S6 Kinase	Cell Signaling Technology	Cat#2708S; RRID: AB_562183
Phospho-S6 Ribosomal Protein (Ser235/236)	Cell Signaling Technology	Cat#4858S; RRID: AB_2146236
S6	Cell Signaling Technology	Cat#2317S; RRID: AB_2238583
RHEB	Cell Signaling Technology	Cat#13879S; RRID: AB_2721022
Phospho-4E-BP1 (Thr37/46)	Cell Signaling Technology	Cat#2855S; RRID: AB_560835
4E-BP1	Cell Signaling Technology	Cat#9644S; RRID: AB_2097841
CREB3L4	Proteintech	Cat No. 13630-1-AP; RRID: AB_2276550
HA	Proteintech	Cat No. 51064-2-AP; RRID: AB_11042321
Ki67	Proteintech	Cat No. 27309-1-AP; RRID: AB_2756525
GAPDH	Proteintech	Cat No. 60004-1-Ig; RRID: AB_2107436

**Bacterial and virus strains**		

Lentivirus-shCREB3L4	Vigene Biology (Jinan, China)	N/A
Lentivirus-shNC	Vigene Biology (Jinan, China)	N/A

**Biological samples**		

Human HCC tissues and matched adjacent tissues	Shandong Provincial Qianfoshan Hospital	N/A
HCC tissue chip	Outdo Biotech (Shanghai, China)	HLiv-HCC180Sur-03

**Chemicals, peptides, and recombinant proteins**		

Rapamycin	MedChemExpress	HY-10219
Puromycin	MedChemExpress	HYB1743A
Sorafenib	MedChemExpress	HY-10201

**Critical commercial assays**		

Cell Counting Kit-8	DOJINDO	CK04
BeyoClick™ EdU Cell Proliferation Kit with Alexa Fluor 488	Beyotime Biotechnology	C0071S
SimpleChIP® Plus Enzymatic Chromatin IP Kit	Cell Signaling Technology	9005
Dual-Luciferase® Reporter Assay System	Promega	E1910

**Deposited data**		

CREB3L4 mRNA expression of HCC	GEPIA	http://gepia.cancer-pku.cn/

**Experimental models: Cell lines**		

Cell line: HUH7	Procell Life Science&Technology	CL0120; RRID:CVCL_0336
Cell line: Hep3B	Procell Life Science&Technology	CL0102; RRID:CVCL_0326

**Experimental models: Organisms/strains**		

Mouse: BALB/c Nude Crlj	Charles River Biotech	409

**Oligonucleotides**		

Primers for qRT-PCR	This paper	N/A
siRNA targeting sequence for RHEB	This paper	N/A

**Recombinant DNA**		

Plasmid: Lentivirus-shCREB3L4	Vigene Biology	N/A
Plasmid: Lentivirus-shNC	Vigene Biology	N/A
Plasmid: CREB3L4	Vigene Biology	N/A

**Software and algorithms**		

SPSS (version 24.0)	IBM	https://www.ibm.com/cn-zh/spss
ImageJ	National Institutes of Health	https://imagej.net
Compusyn	ComboSyn, Inc.	https://www.combosyn.com/

### Resource availability

#### Lead contact

Further information and request for resources and reagents should be directed to and will be fulfilled by the Lead Contact, Xiaomin Ma (15910098683@163.com); Sanyuan Hu(drsanyuanhu@163.com).

#### Materials availability

This study did not generate new unique reagents.

#### Data and code availability

• All data generated or analyzed during this study are included in this article. Further enquiries can be directed to the lead contact.
• This paper does not report original code.
• Any additional information required to reanalyze the data reported in this paper is available from the lead contact upon request.

### Experimental model and study participant details

#### Clinical samples

One hundred and three pairs of HCC tissues and matched adjacent tissues were obtained from the HCC patients admitted to our hospital for treatment. HCC patients underwent chemotherapy, radiotherapy and target therapy were excluded from this study. Patients’ characteristics were summarized in Table S1. Cohort 1 involved 83 patients, and their HCC tissue chips were prepared by Outdo Biotech (Shanghai, China). Cohort 2 involved 20 HCC patients admitted to our hospital for treatment. All patients signed informed consent forms and all experimental protocols were consistent with Declaration of Helsinki. The study protocols were approved by the Ethics Committee of Shandong Provincial Qianfoshan Hospital (2021-S202).

#### Mice

Male BALB/c nude mice (5 weeks old, 15-20g) purchased from Charles River Biotech (Beijing, China) were raised under specific pathogen-free conditions (temperature 24°C; relative humidity 40–60%; 12-h light/dark cycles). The study protocols were approved by the Ethics Committee of Shandong Provincial Qianfoshan Hospital [2021-S199].

#### Cell lines

HUH7 (RRID:CVCL_0336) and Hep3B (RRID:CVCL_0326) cells purchased from ATCC (Shanghai, USA) were cultured in DMEM medium (Beyotime, Hangzhou, China) and MEM (Basalmedia, Shanghai, China), respectively. The medium was supplemented with 1% streptomycin and penicillin (Sigma-Aldrich) and 10% fetal bovine serum (FBS, Sigma-Aldrich), which was cultured in a humidified incubator containing 5% CO_2_ at 37°C. All cell lines have been authenticated in the past three years. Cell line authentication process was conducted by CellCook Biotech (Guangzhou, China). All the experiments were conducted using mycoplasma-free cells.

### Method details

#### Cell transfection

Short hairpin RNAs (shRNAs) plasmid against CREB3L4 purchased from Vigene Biology (Jinan, China) was utilized to induce CREB3L4 silencing, while the HA-CREB3L4 plasmid was utilized to induce CREB3L4 over-expression. The transfection was performed using Lipofectamine 2000 (Invitrogen, Carlsbad, CA, USA). HCC cells transfected with shCREB3L4 or HA-CREB3L4 related vector plasmids served as mock control.

#### Real-Time PCR

RNAfast200 kit (Fastagen, Shanghai, China) was utilized to extract total RNA from clinical samples and HCC cell lines. The cDNA was synthesized using PrimeScripttm^RT^ reagent Kit with gDNA Eraser (Takara, Shiga, Japan) according to the manufacturer’s instructions. Quantitative PCR was performed using FastStart Universal SYBR Green Master on an Applied Roche LightCycler 480 II system.

#### Cell viability and colony formation assays

About 24 h after transfection, cells (1 × 10^4^ cells) were seeded into 96-well plates. Then cell counting kit-8 (CCK-8) assay and EdU assay were performed to test the cellular proliferation at 0, 24 h, 48 h and 72 h, respectively. Then we evaluated the colony formation ability of HCC cells. Briefly, 24 h after transfection, the transfected HCC cells (1 × 10^3^ cells per well) were seeded into 6-well plates. After culturing for additional 14 days, cells were fixed and stained with 1% crystal violet (Solarbio, Beijing, China), followed by counting and normalization of the number of colonies to corresponding control cells.

#### Chromatin immunoprecipitation assay (ChIP)

Chromatins extracted from transfected HUH7 cells were fixed and immunoprecipitated per the manufacturer’s instructions (9005, Cell Signaling Technology). Then anti-HA (2 μg) was used to purify the chromatins. The binding between CREB3L4 and *RHEB* promoter was assessed by PCR. Sonicated DNA fragments used for immunoprecipitation showed a length of 200 bp based on ethidium bromide gel electrophoresis.

#### Dual-luciferase reporter assay

HUH7 cells were seeded overnight in 12-well plates, followed by co-transfection with firefly luciferase plasmid (0.5 μg) expressing the wild-type *RHEB* promoter, or *RHEB* mutation promoter (0.5 μg) and Renilla luciferase expressing plasmid (0.01 μg, Vigene Biology, Jinan, China). About 48 h after transfection, luciferase activity was performed to the harvested cells using a Dual-Luciferase Reporter Assay System (E1910, Promega), according to the manufacturer’s instructions. Firefly luciferase data were then normalized to Renilla luciferase data that were presented as the fold change relative to the control. In each group, six replicates were set for the dual-luciferase reporter assay, and each test was performed at least in triplicate. A sequence with a length of 600 bp (from −1 to −600 position) was linked to the pGL3-basic vector, based on the SacI-XhoI restriction sites. Additionally, mutation vectors were also constructed to mutate the two site sequences into ‘TTGTTATATTAA’ and ‘CTAATTTGTTAT’. Similarly, these sequences were linked to the pGL3-basic vector, to determine the expression of luciferase in the wild type and mutation.

#### Construction of stable infection of HUH7 cells with Lentivirus (LV)-shCREB3L4 plasmid

LV-shCREB3L4 and LV-shNC plasmids were purchased from Vigene Biology (Jinan, China). A stable CREB3L4 knockdown HUH7 cell line was established using viral loads with a viral MOI of 10 or 20. The results showed that cells were transfected for 48 h with the highest transfection efficiency in the presence of a MOI of 20 (Figure S1A). After 72 h culture, cells were stimulated with puromycin (5 μg/mL), and puromycin was replaced every 3 days for 2 weeks to obtain the stable CREB3L4 knockdown HUH7 cell line.

#### Introduction of xenograft tumor model

Then the animals were randomly divided into two group (n = 6 per group), underwent subcutaneous injection of LV-shCREB3L4 plasmid transfected HUH7 cells (8 × 10^6^ cells/100 μL) (designated as shNC group) or LV-shNC plasmid transfected HUH7 cells (8 × 10^6^ cells/100 μL) (designated as shCREB3L4 group), respectively. We also propagated it *in vitro* for the same duration, and then regularly detected the expression of mRNA (Figure S1B). After the *in vivo* experiment, the target gene mRNA was detected and combined with the Western blot of tumor samples to ensure high transfection efficiency (Figure S1C). The tumor volume and tumor weight were determined in each group in the presence of CREB3L4 silencing according to the previous description.^34^

#### Chemosensitivity assay

Both *in vitro* and *in vivo* tests were performed to analyze the effects of CREB3L4 on chemosensitivity. For the *in vitro* test, HUH7 cells and Hep3B cells were subjected to sorafenib with a concentration of 0, 0.01 μM, 0.1 μM, 1 μM, 10 μM, 100 μM about 48 h after transfection. Upon the selection of IC_50_, the cells were treated with 10 μM sorafenib for 48 h to determine the chemosensitivity assay by CCK-8 assay. For the *in vivo* test, upon presence of visible tumors, animals in the shCREB3L4 group and shNC groups were divided into the following groups: (a) shNC-PBS group (n = 4), treated with PBS solution; (b) shNC-Sorafenib group (n = 4), treated with sorafenib (5 mg/kg); (c) shCREB3L4-PBS group (n = 4), treated with PBS; and (d) shCREB3L4-Sorafenib group, treated with sorafenib (5 mg/kg). Each treatment was given every 4 days. The mice were sacrificed 5 weeks after treatment in each group, followed by isolation of tumor masses for further analysis.

#### Immunohistochemistry and Western blot

IHC was performed based on the tissue chip and the tumor mass from the animals. Western blot was conducted based on 20 HCC samples, tumor mass from animals and the cell lines. Both IHC and Western blot assays were carried out according to the previous study.^35^ IHC data were quantitatively analyzed using ImageJ software as previously described.^36^^,^^37^

### Quantification and statistical analysis

SPSS (version 24.0) was used for the data analysis. All data were represented as mean ± standard deviation. The one-way ANOVA and/or unpaired Student’s *t* test was used for the data comparison. Log Rank test was performed for the survival analysis. All tests were two-tailed, and a *P* of less than 0.05 was considered to be statistically significant. p values are denoted in figures as follows: ∗p < 0.05, ∗∗p < 0.01, ∗∗∗p < 0.001.

### Additional resources

Not applicable.
