# Supplementary material for: CREB3L4 promotes hepatocellular carcinoma progression and decreases sorafenib chemosensitivity by promoting RHEB-mTORC1 signaling pathway
Source: iScience. 2024 Jan 9;27(2):108843. doi: 10.1016/j.isci.2024.108843 (PMC10831937; doi:10.1016/j.isci.2024.108843)
Supplement: Document S1. Figures S1–S5, Table S1, and Data S1–S17 [file mmc1.pdf]

iScience, Volume *27*

Supplemental information

CREB3L4 promotes hepatocellular carcinoma progression and decreases sorafenib chemosensitivity by promoting RHEB-mTORC1 signaling pathway

Zhengchen Jiang, Bowen Shi, Yun Zhang, Tianming Yu, Yang Cheng, Jiankang Zhu, Guangyong Zhang, Mingwei Zhong, Sanyuan Hu, and Xiaomin Ma

Supplementary Table 1 Clinicopathological characteristics of HCC patients，related to STAR Method (Clinical samples)

**Characteristics**	**Cohort 1 (n=83)**	**Cohort 2 (n=20)**
Gender		
Male	71(85.5%)	16(80.0%)
Female	12(14.5%)	4(20.0%)
Age		
<60 yrs	59(71.1%)	12(60.0%)
≥60 yrs	24(28.9%)	8(40.0%)
Liver cirrhosis history		
Yes	18 (21.6%)	10(50.0%)
No	65 (78.4%)	10(50.0%)
TNM stages		
I	7(8.4%)	4(20.0)
II	47(56.7%)	9(45.0%)
III	29(34.9%)	7(35.0%)
IV	0(0%)	0(0%)
Pathological staging		
I	16(1.1%)	8(40.0%)
II	59(45.6%)	10(50.0%)
III	8(53.3%)	2(10.0%)

Cohort 1 was derived from the HCC tissue chip purchased from Outdo Biotech (Shanghai, China). Cohort 2 was derived from patients with HCC admitted to our hospital for treatment.

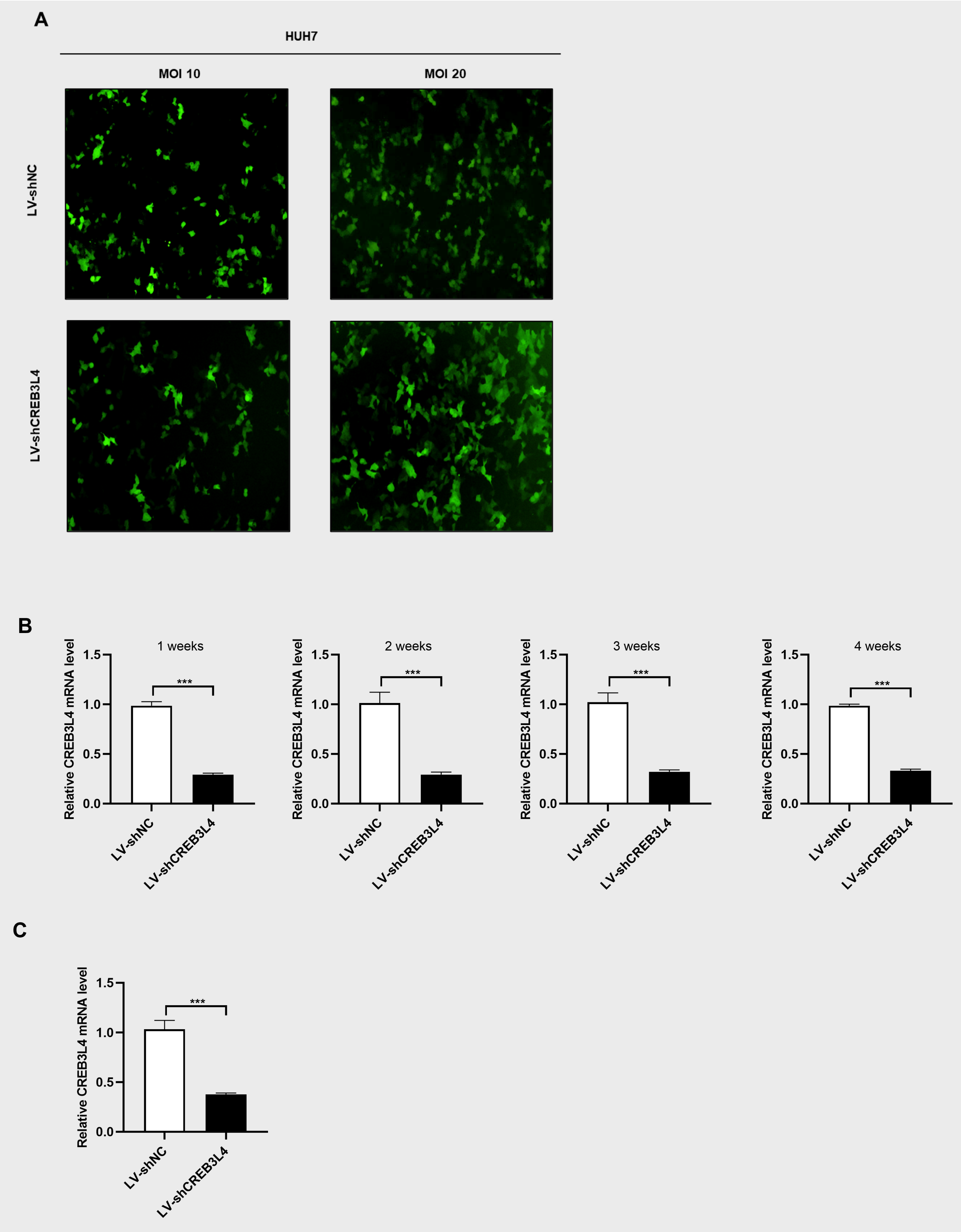

Supplementary Figure 1 Construction of stable infection of HUH7 cells with Lentivirus (LV)-shCREB3L4 plasmid, related to STAR Method (Construction of stable infection of HUH7 cells with Lentivirus (LV)-shCREB3L4 plasmid).

(A) Lentiviral transfection of HUH7 cell lines was performed using a MOI of 10 or 20 viral loads, and the fluorescence intensity was observed under the microscope. (B) The qRT-PCR was performed to detect the effective knockdown of HUH7 cells infected with LV-shCREB3L4 after infected for 4 weeks. (C) The qRT-PCR was performed to detect the effective knockdown of dissected tumors.

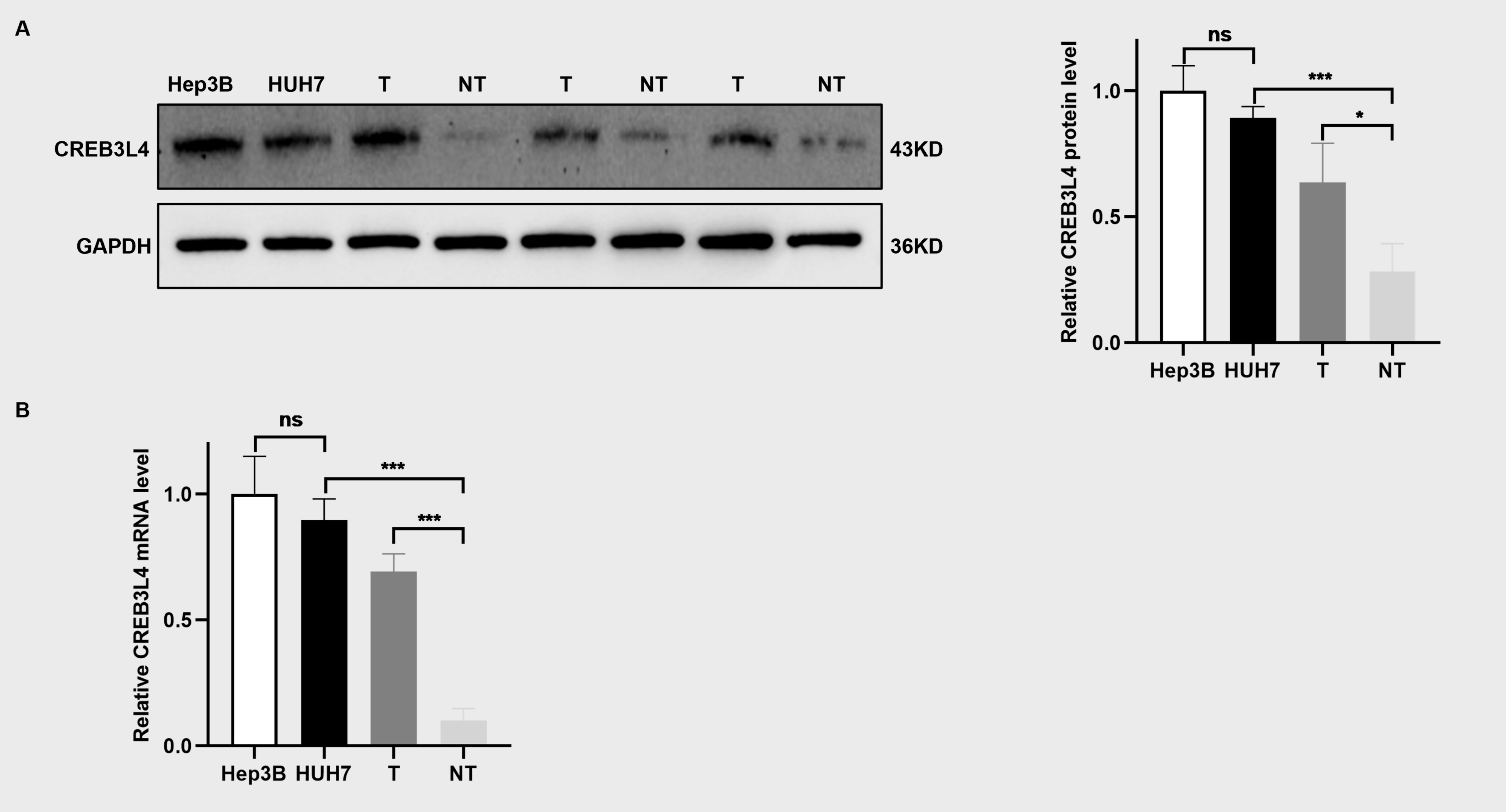

Supplementary Figure 2 Basal level of CREB3L4 in HCC cell lines and clinic samples, related to Figure 1. (A) Western blot assay was conducted to measure the relative expression of the CREB3L4 protein in HUH7, Hep3B cells, tumor tissues (T) and the normal tissues (NT). Western blot bands were analyzed by ImageJ software. (B) qRT-PCR analysis was conducted to measure the relative expression of the CREB3L4 mRNA in HUH7, Hep3B cells, tumor tissues (T) and the normal tissues (NT).

A

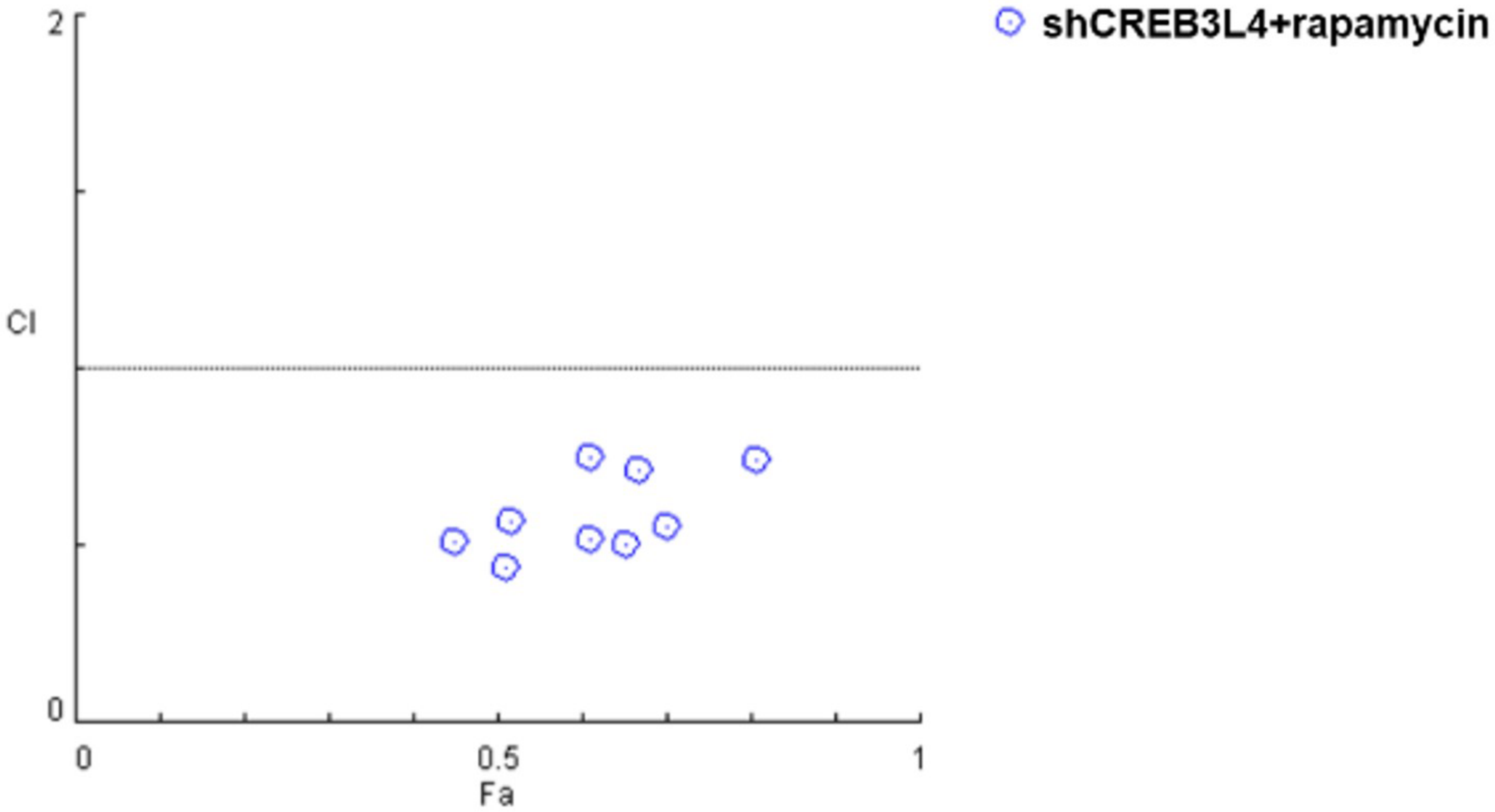

Supplementary Figure 3 Combination index values of rapamycin, related to Figure 3. (A) HUH7 cells (1 × 104 cells/well) were transfected with different dosages of shCREB3L4 plasmid (0.5 µg/ml, 1 µg/ml, and 2 µg/ml) and different dosages of rapamycin (20 nM, 40 nM, and 80 nM). Cellular viability was detected by CCK-8 assay after treating with shCREB3L4 and rapamycin for 48 hr. Combination index values were calculated by Compusyn software, and drug interaction was indicated as synergism (CI < 0.9), additivity (0.9 < CI < 1.1) or antagonism (CI > 1.1).

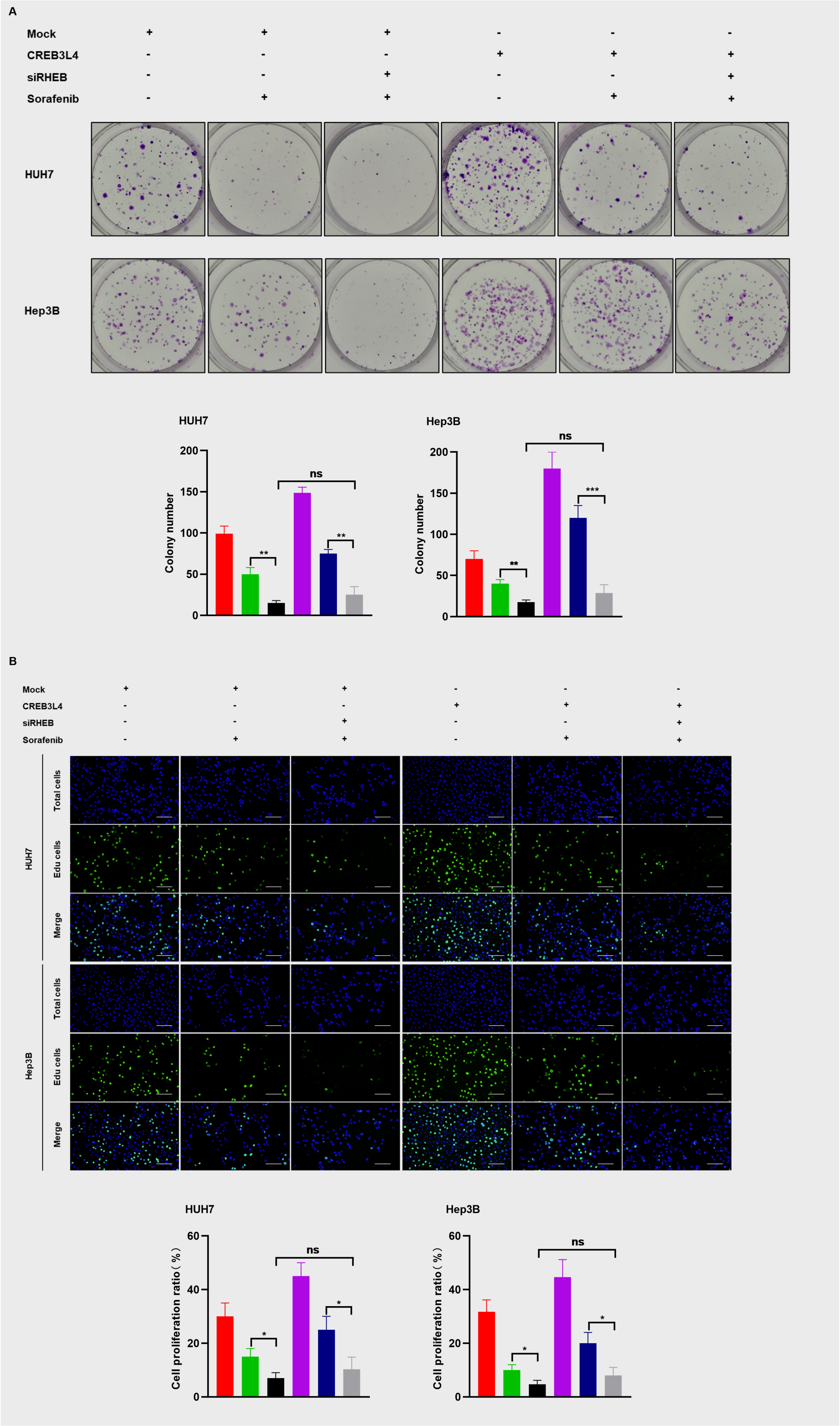

Supplementary Figure 4 RHEB knockdown reversed the promoting effects of CREB3L4 on the proliferation of HUH7 and Hep3B cells, related to Figure 4. (A) HUH7 and Hep3B cells were transfected with CREB3L4 overexpressing plasmid or siRHEB. Approximately 24 hr after transfection, cells were treated with sorafenib (10 μM) for 48 hr. Colony formation assay was performed to detect the proliferation of the cells. (B) Cell proliferation was detected by EdU assays. * P <0.05, **P <0.01, ***P <0.001 for statistical analysis of the indicated groups.

A

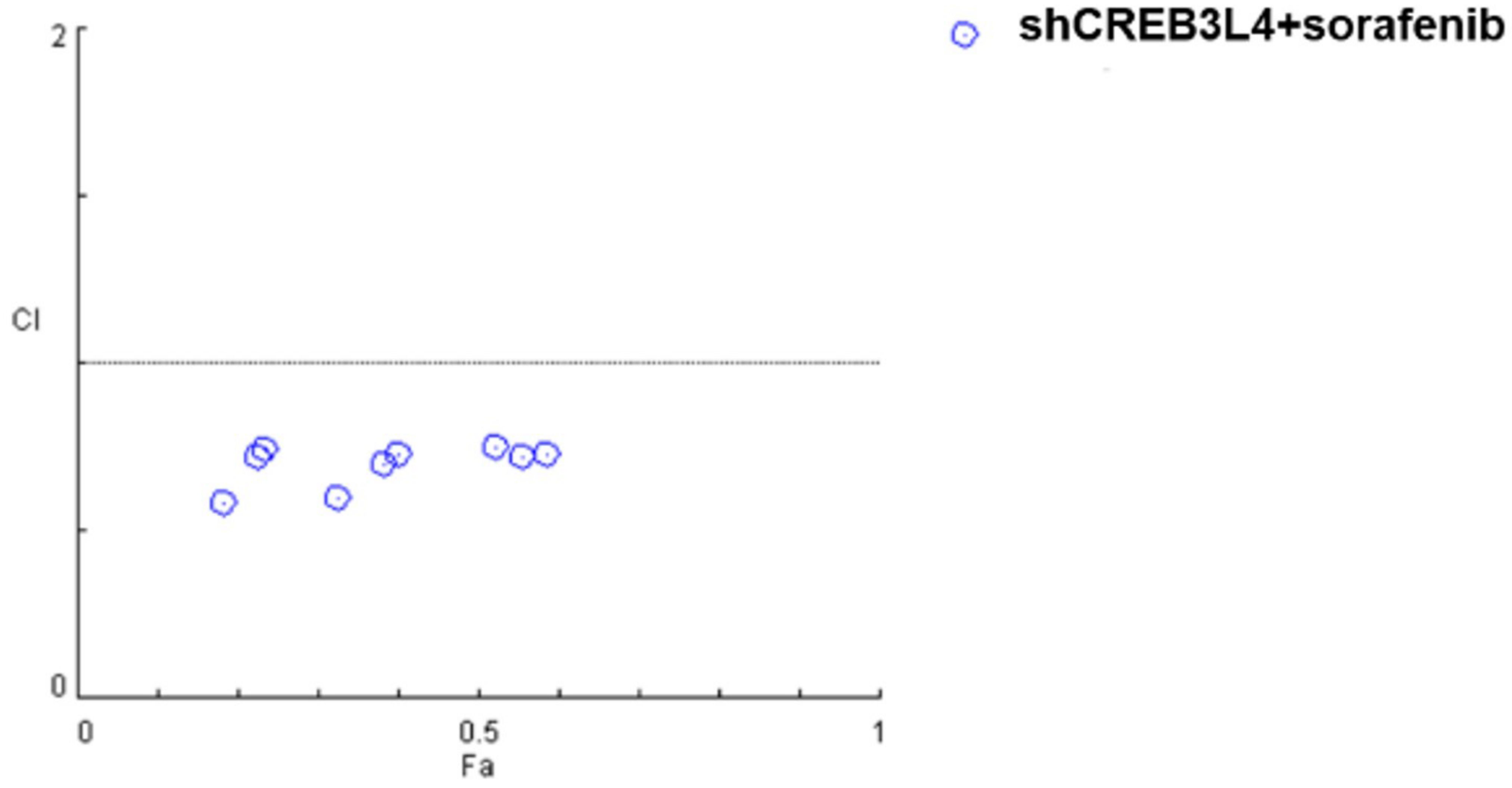

Supplementary Figure 5 Combination index values of sorafenib, related to Figure 5.

(A) HUH7 cells (1 × 104 cells) were transfected with different dosages of shCREB3L4 plasmid (0.5 µg/ml, 1 µg/ml, and 2 µg/ml) and different dosages of sorafenib (5 µM, 10 µM, and 20 µM). Cell viability was detected by CCK-8 assay after treating with shCREB3L4 and sorafenib for 48 hr. Drug interaction was indicated as synergism (CI < 0.9), additivity (0.9 < CI < 1.1) or antagonism (CI > 1.1).

CREB3L4
43KD

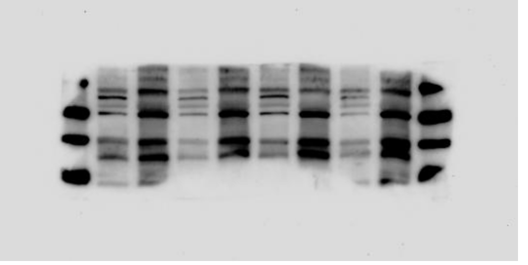

GAPDH
36KD

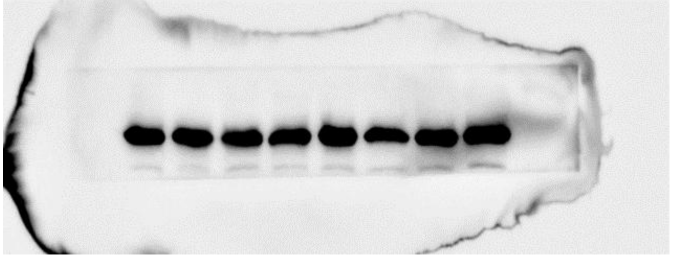

Data S1 Original images of Western blot, related to Figure 1E.

HUH7
CREB3L4
43KD

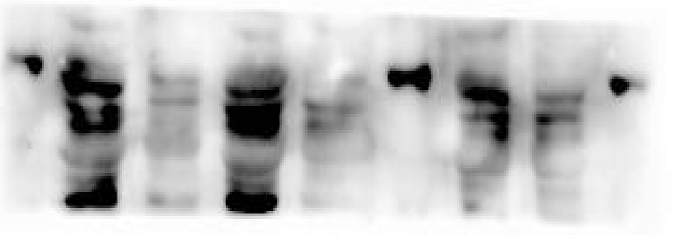

HUH7
GAPDH
36KD

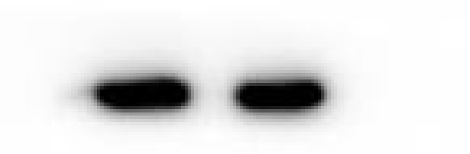

Hep3B
CREB3L4
43KD

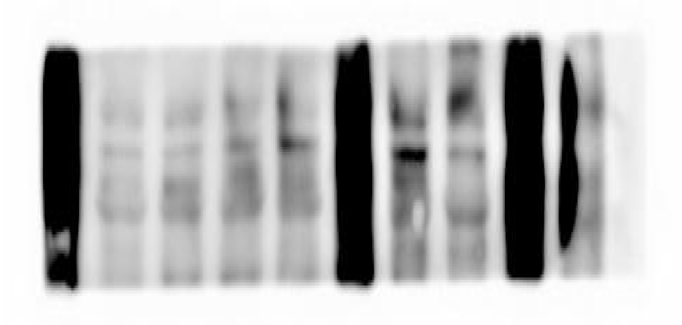

Hep3B
GAPDH
36KD

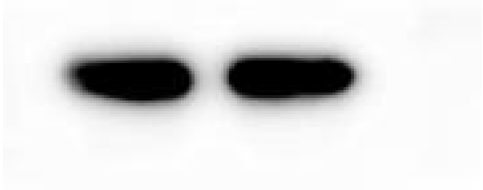

Data S2 Original images of Western blot, related to Figure 2C.

HUH7
HA-
CREB3L4
43KD

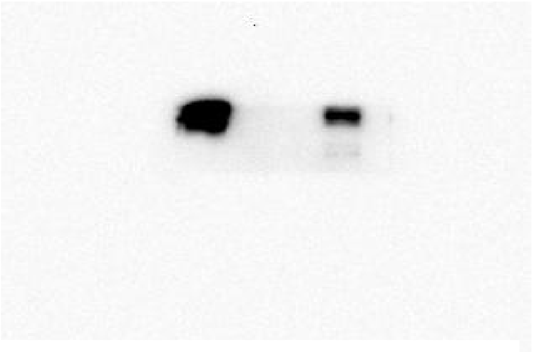

HUH7
GAPDH
36KD

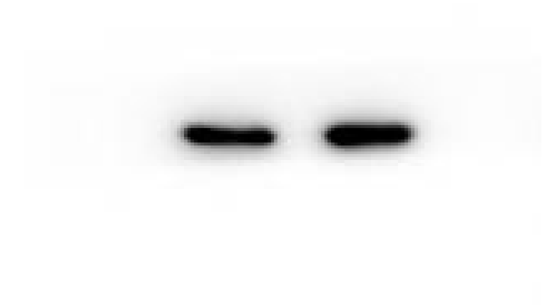

Hep3B
HA-
CREB3L4
43KD

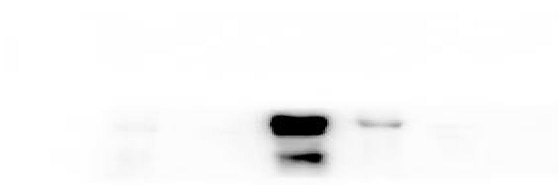

Hep3B
GAPDH
36KD

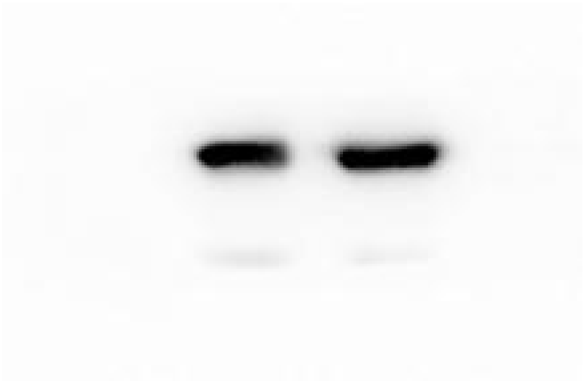

Data S3 Original images of Western blot, related to Figure 2D.

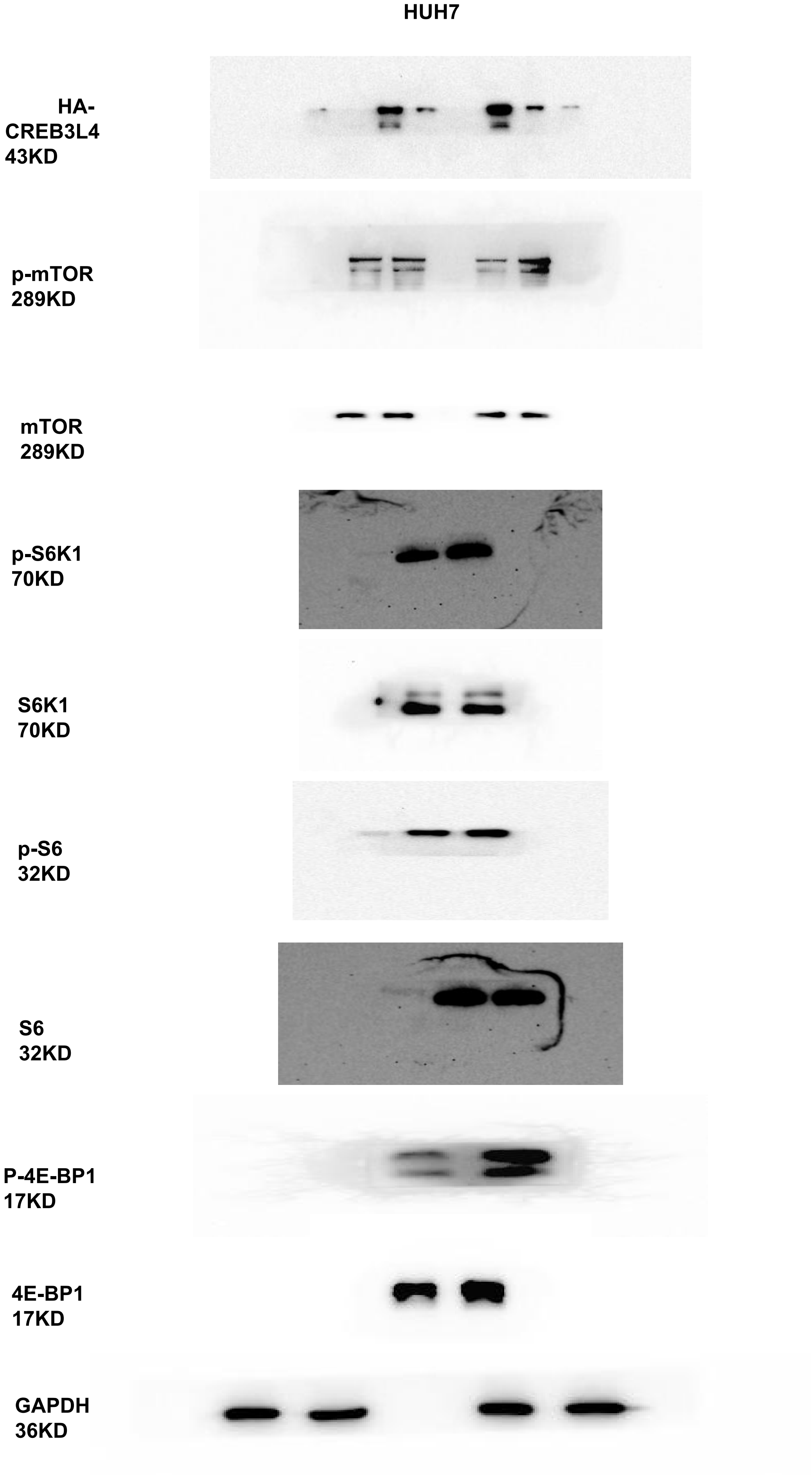

Data S4 Original images of Western blot, related to Figure 3A.

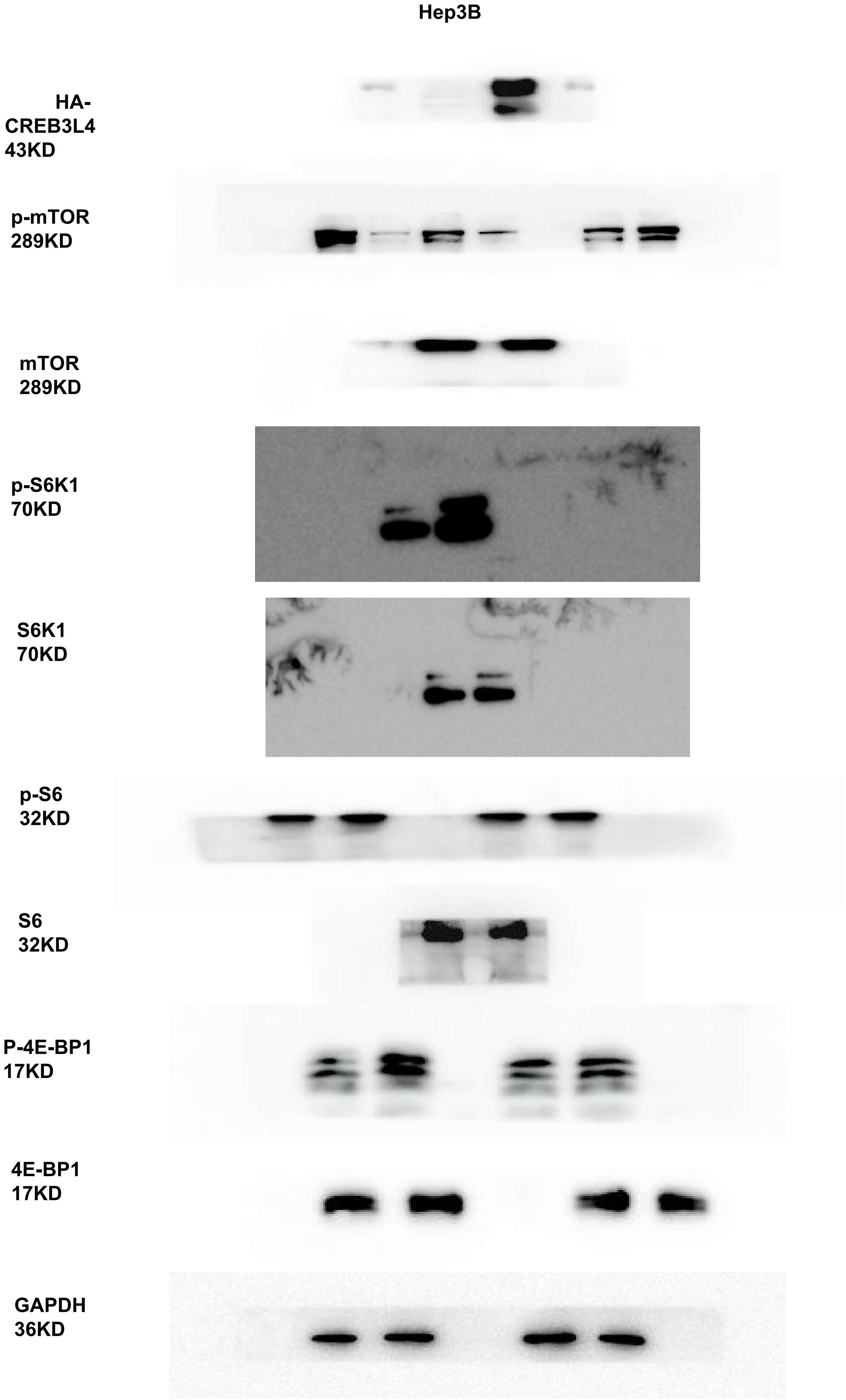

Data S5 Original images of Western blot, related to Figure 3A.

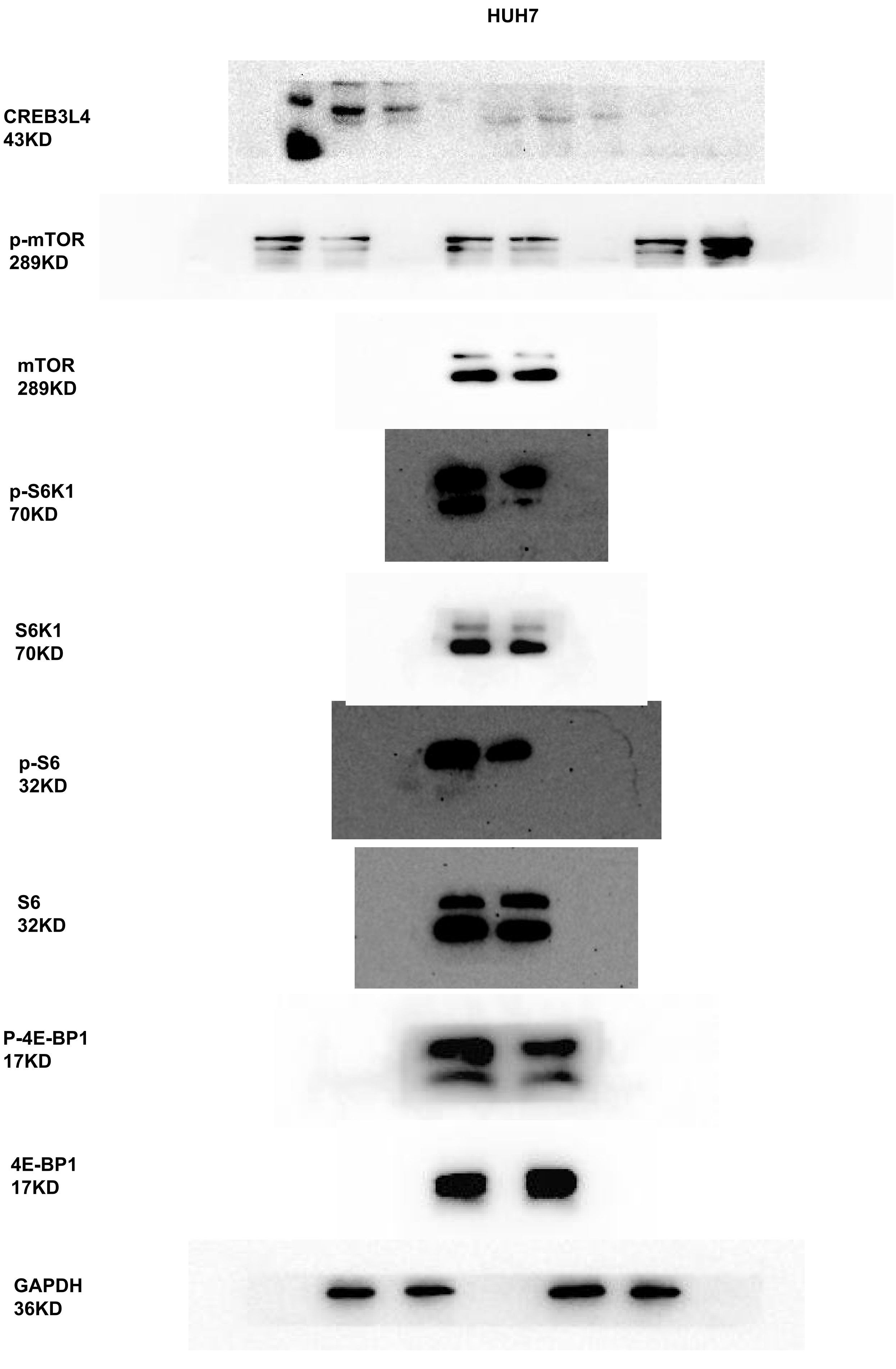

Data S6 Original images of Western blot, related to Figure 3B.

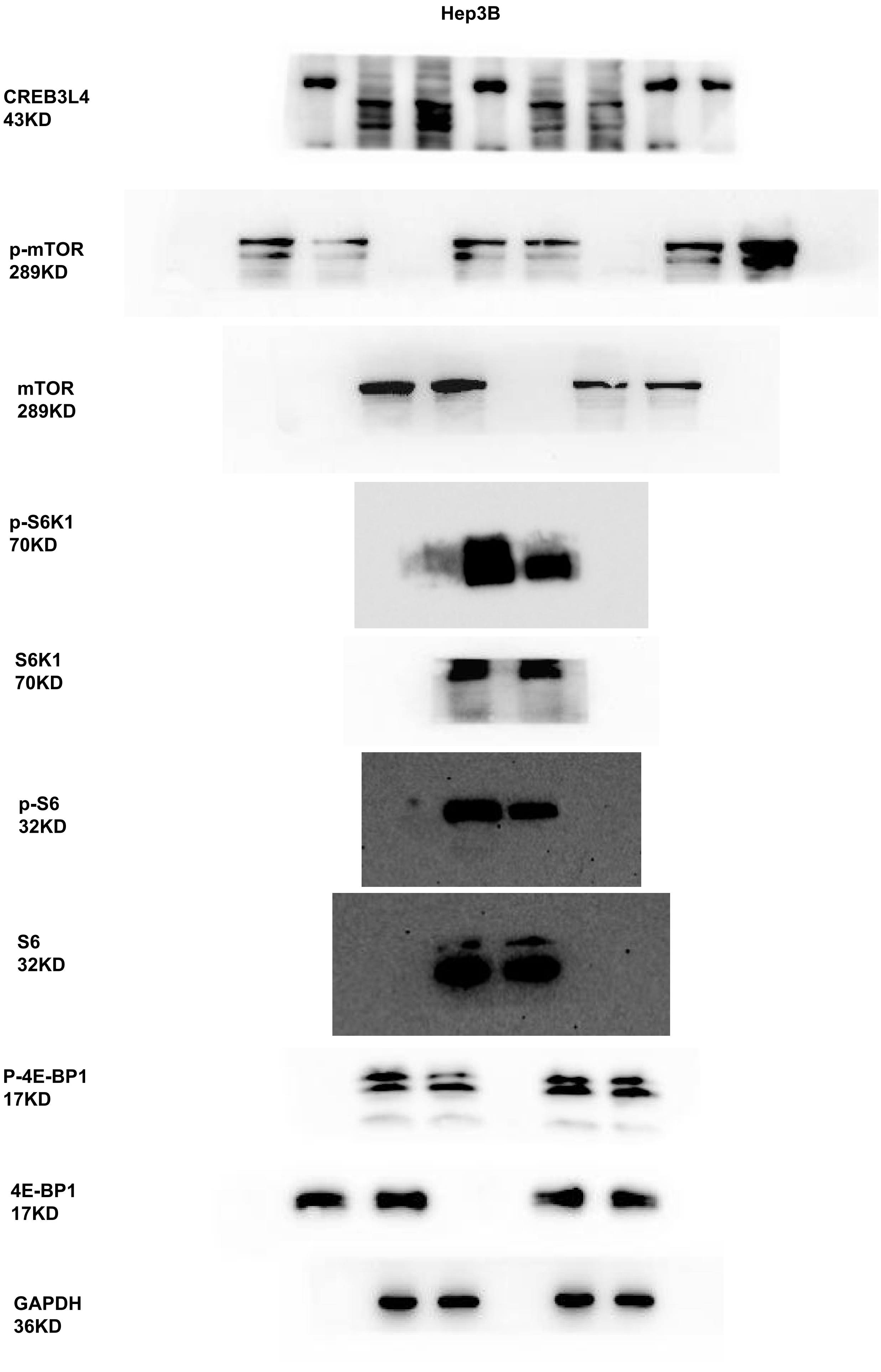

Data S7 Original images of Western blot, related to Figure 3B.

HUH7

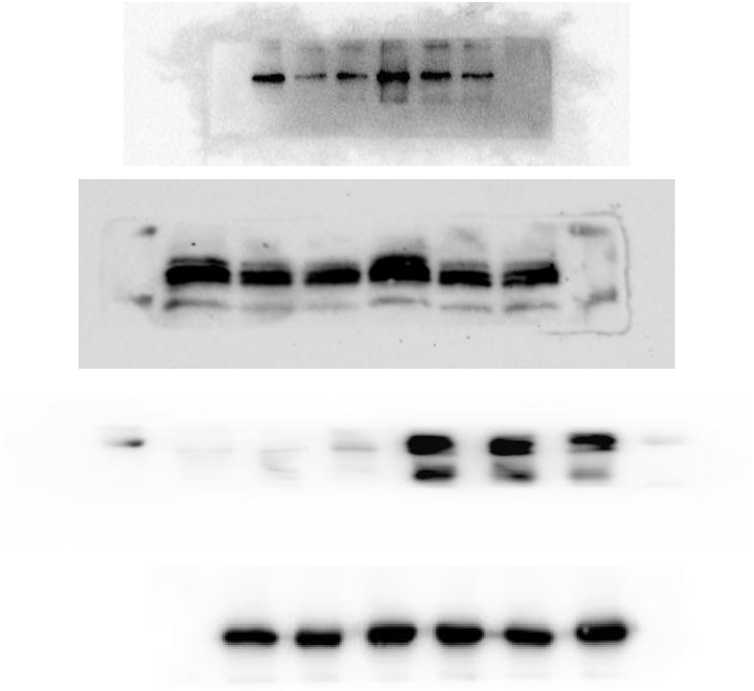

p-mTOR
289KD

p-S6
32KD

CREB3L4
43KD

GAPDH
36KD

Hep3B

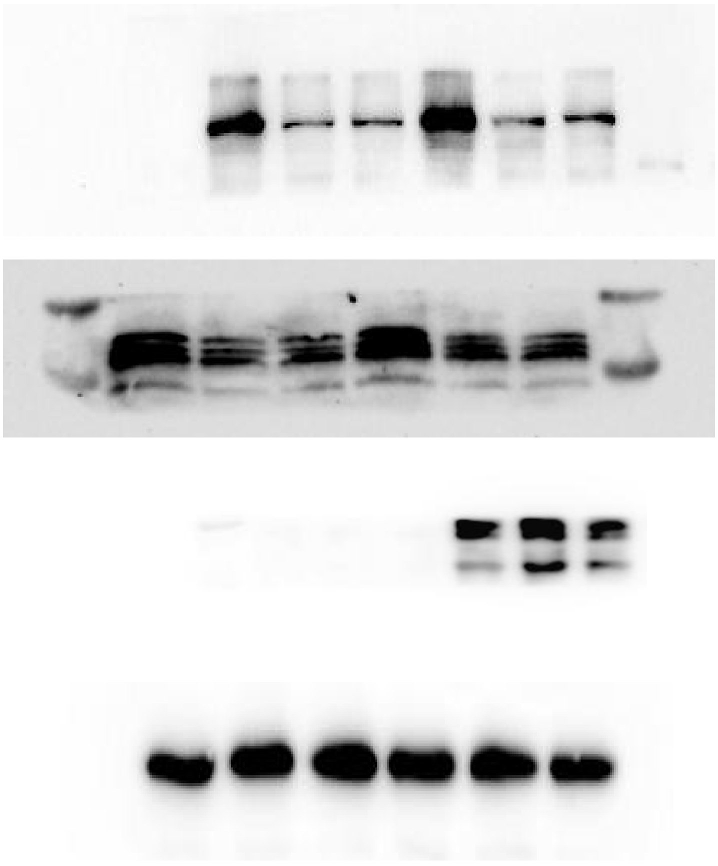

p-mTOR
289KD

p-S6
32KD

CREB3L4
43KD

GAPDH
36KD

Data S8 Original images of Western blot, related to Figure 3C.

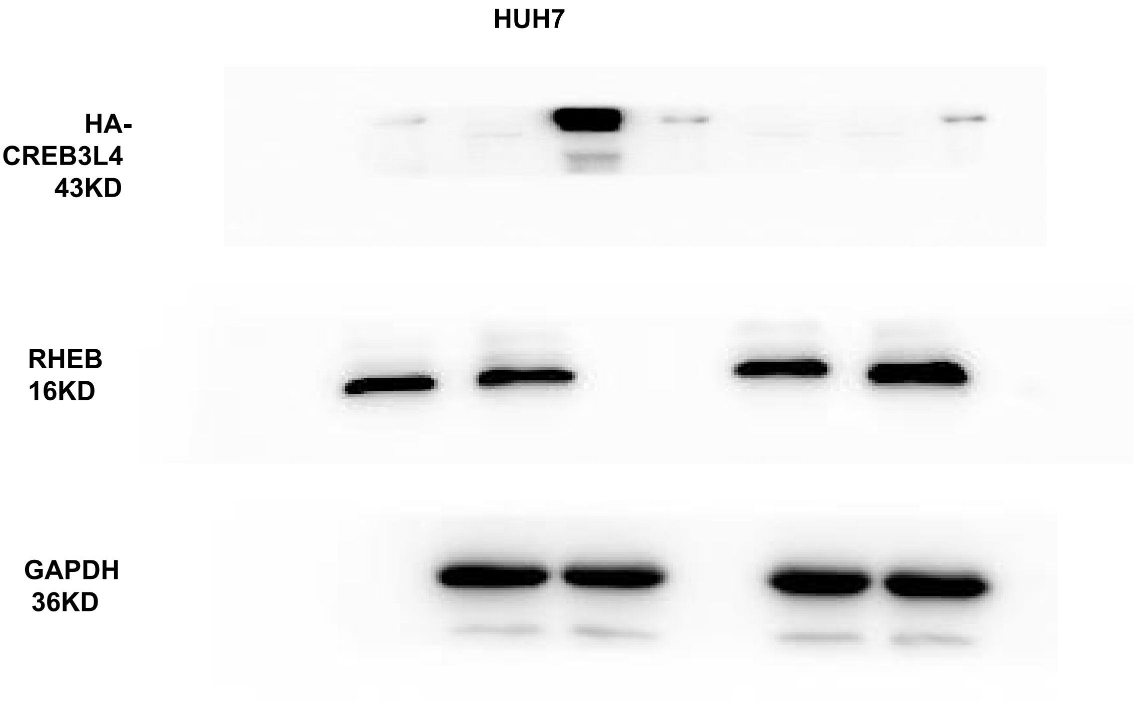

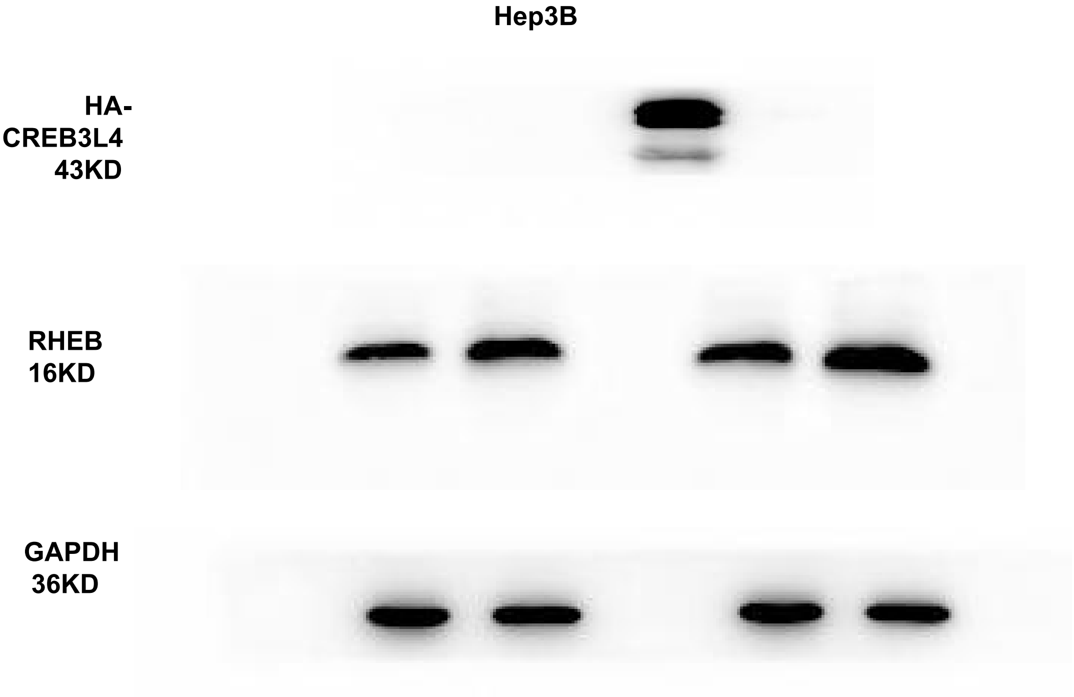

Data S9 Original images of Western blot, related to Figure 4C.

HUH7

CREB3L4
43KD

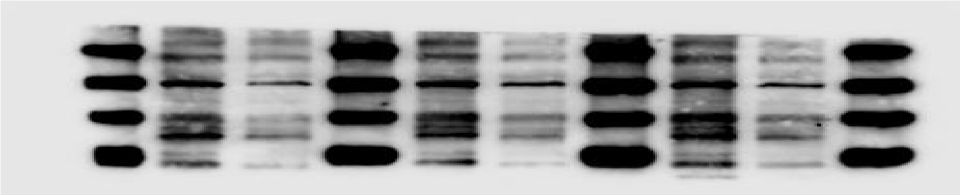

RHEB
16KD

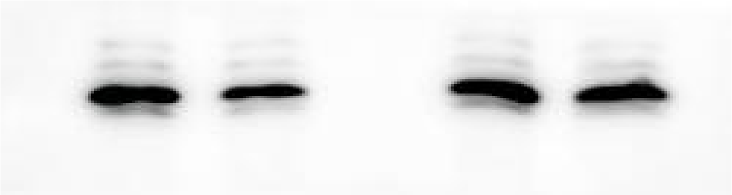

GAPDH
36KD

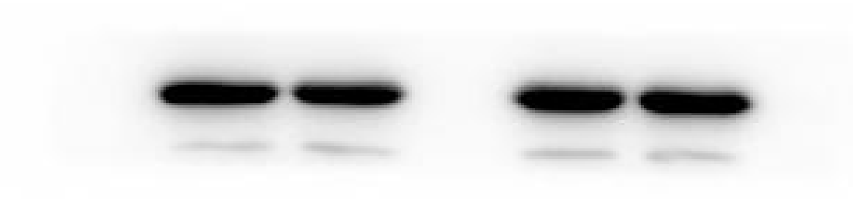

Hep3B

CREB3L4
43KD

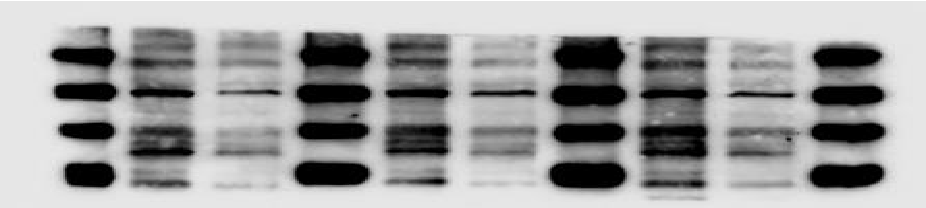

RHEB
16KD

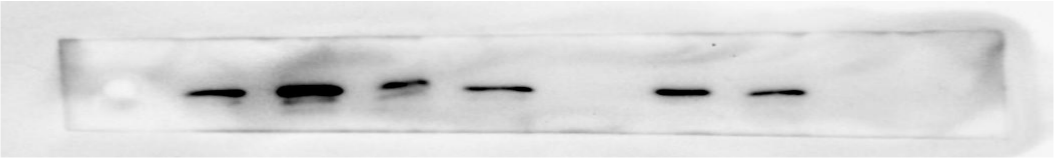

GAPDH
36KD

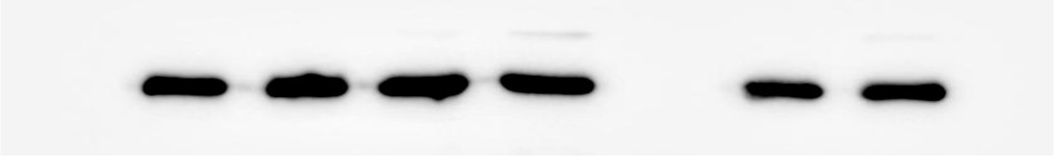

Data S10 Original images of Western blot, related to Figure 4D.

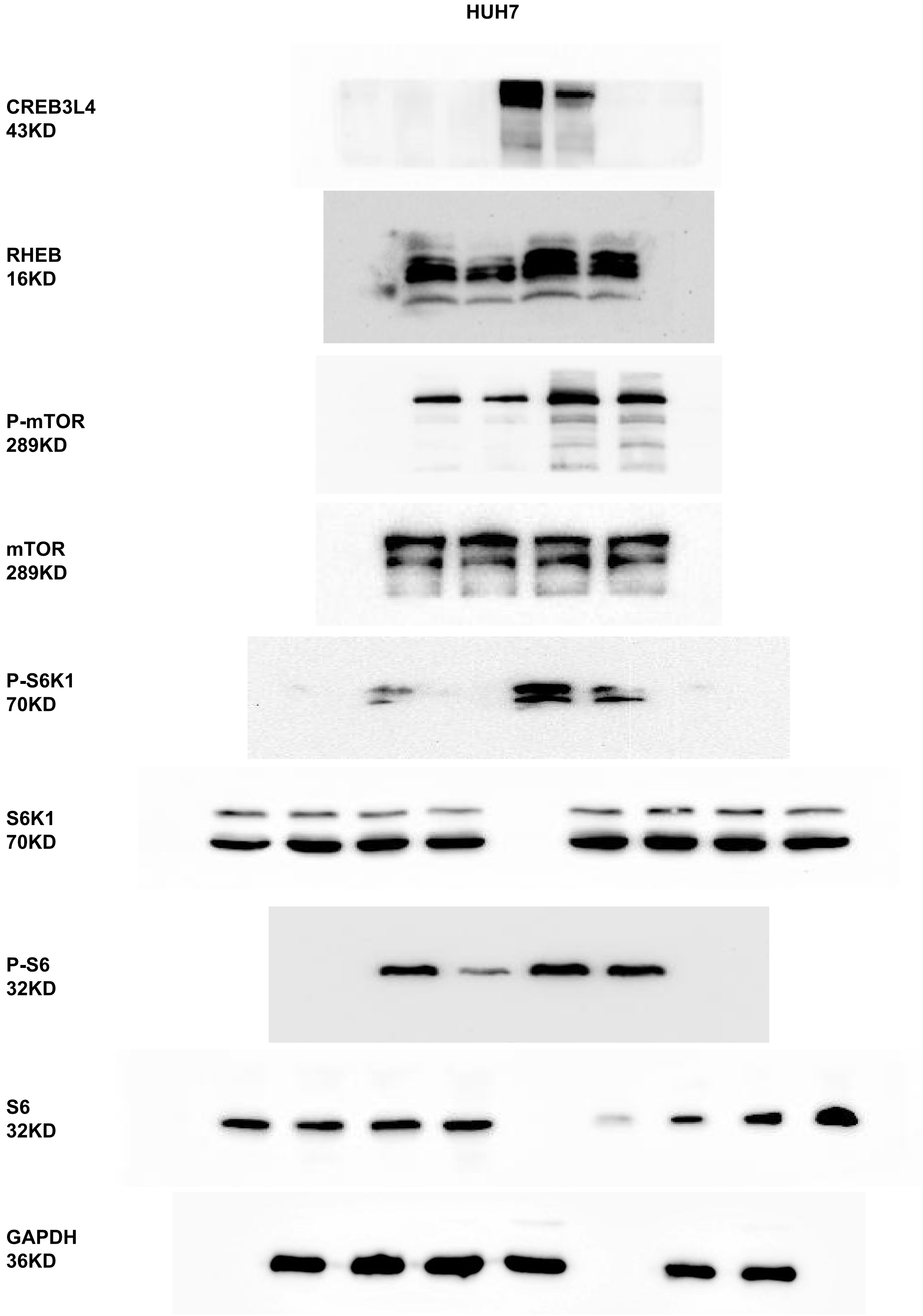

Data S11 Original images of Western blot, related to Figure 5G.

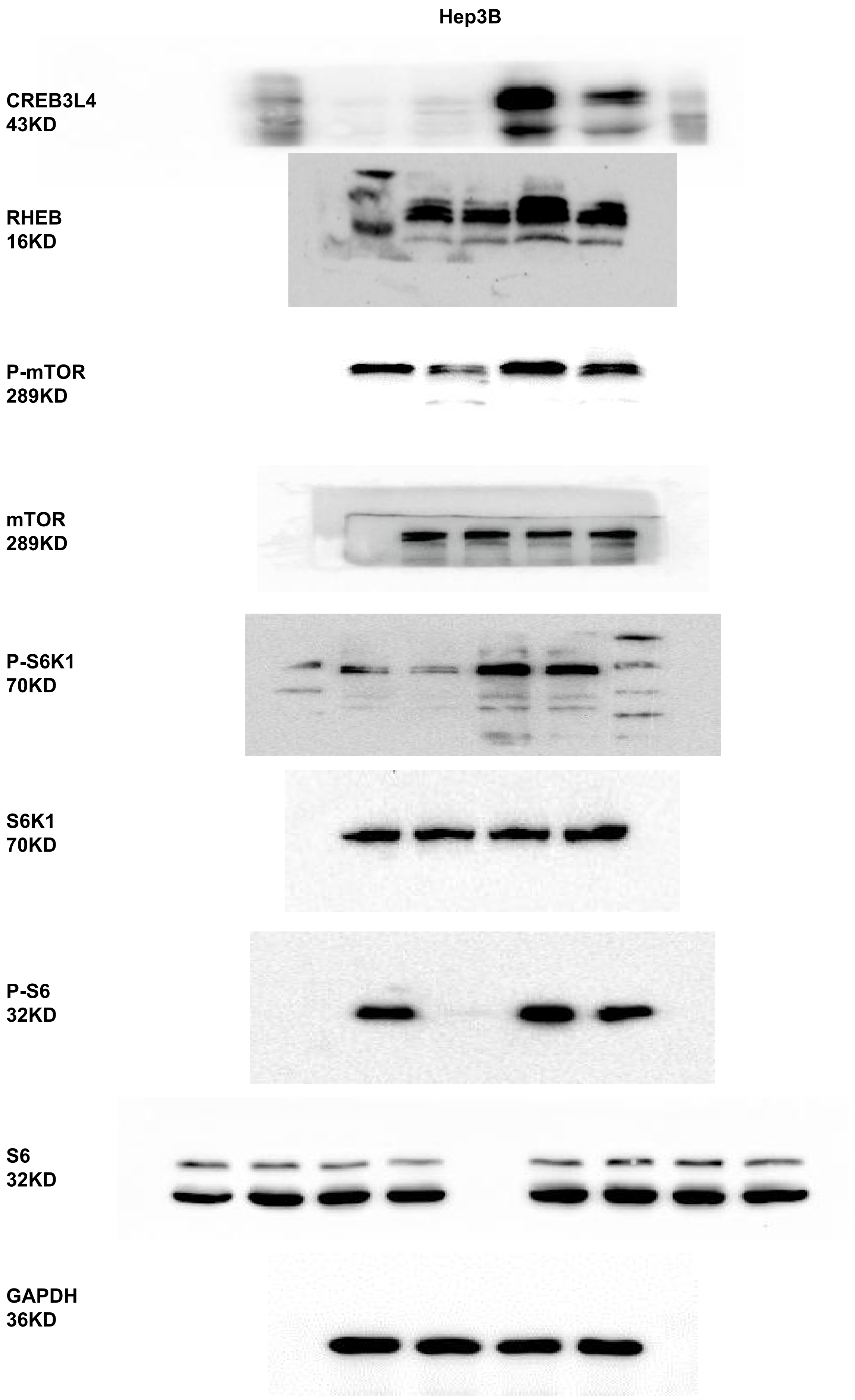

Data S12 Original images of Western blot, related to Figure 5G.

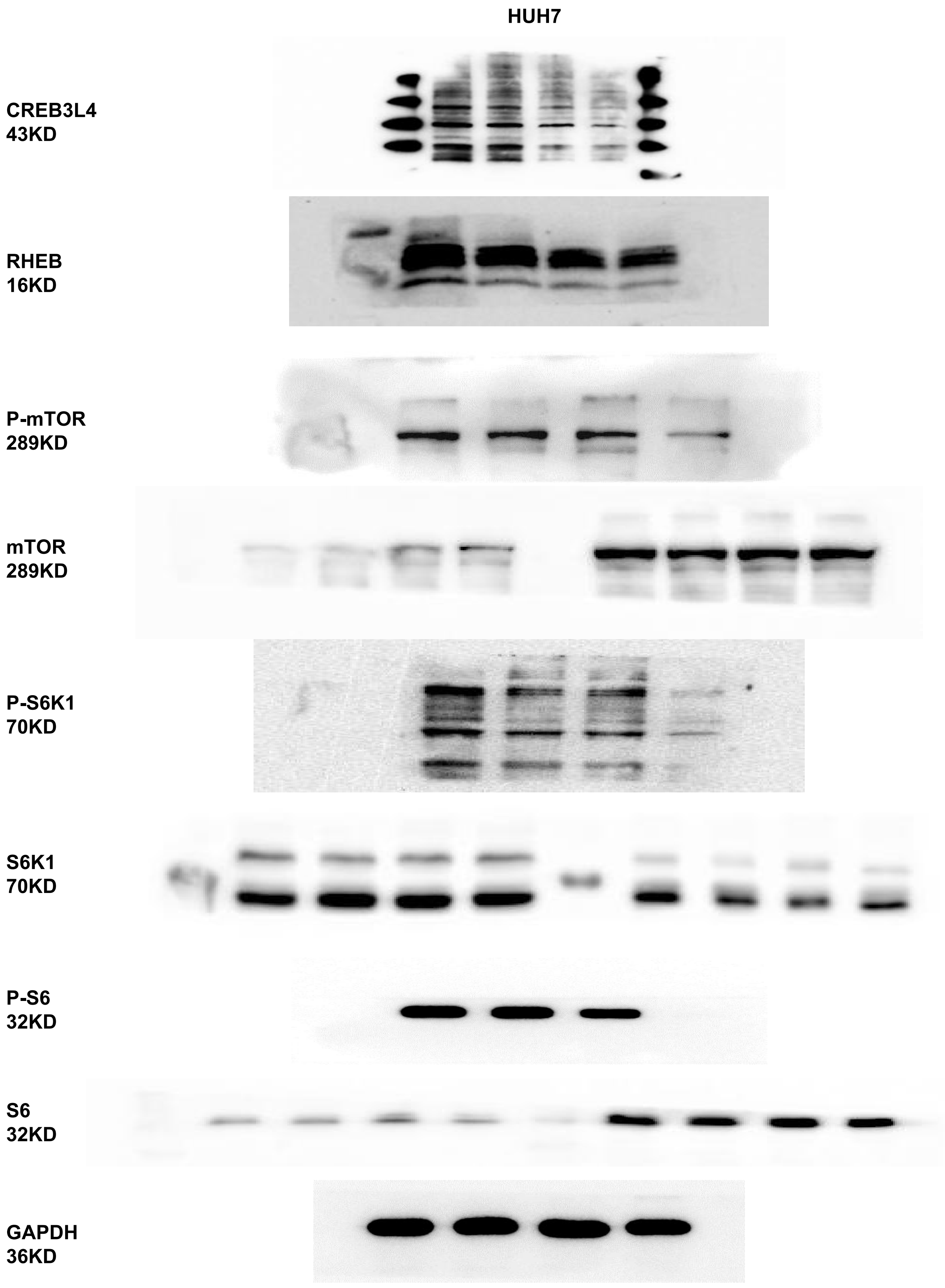

Data S13 Original images of Western blot, related to Figure 5H.

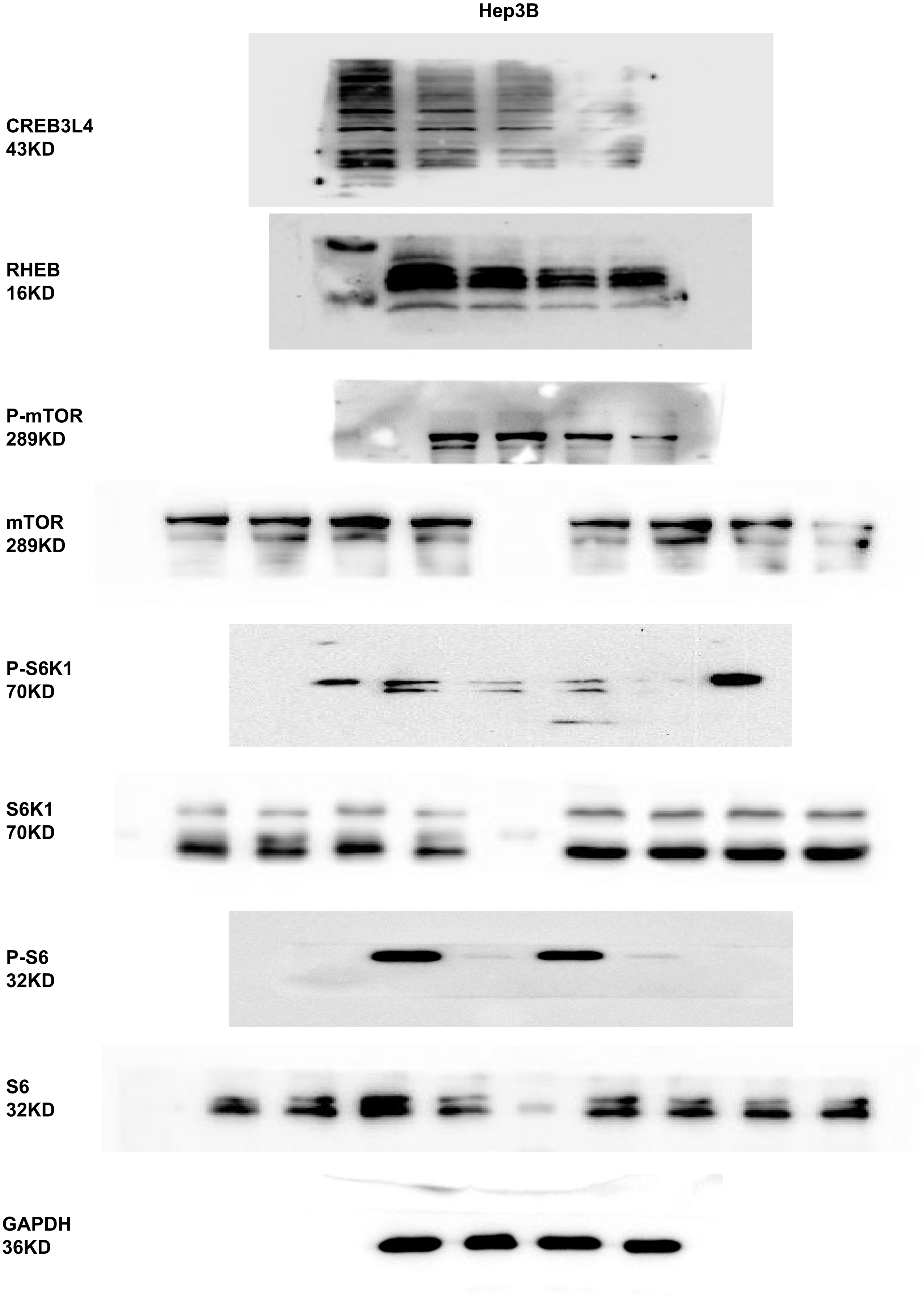

Data S14 Original images of Western blot, related to Figure 5H.

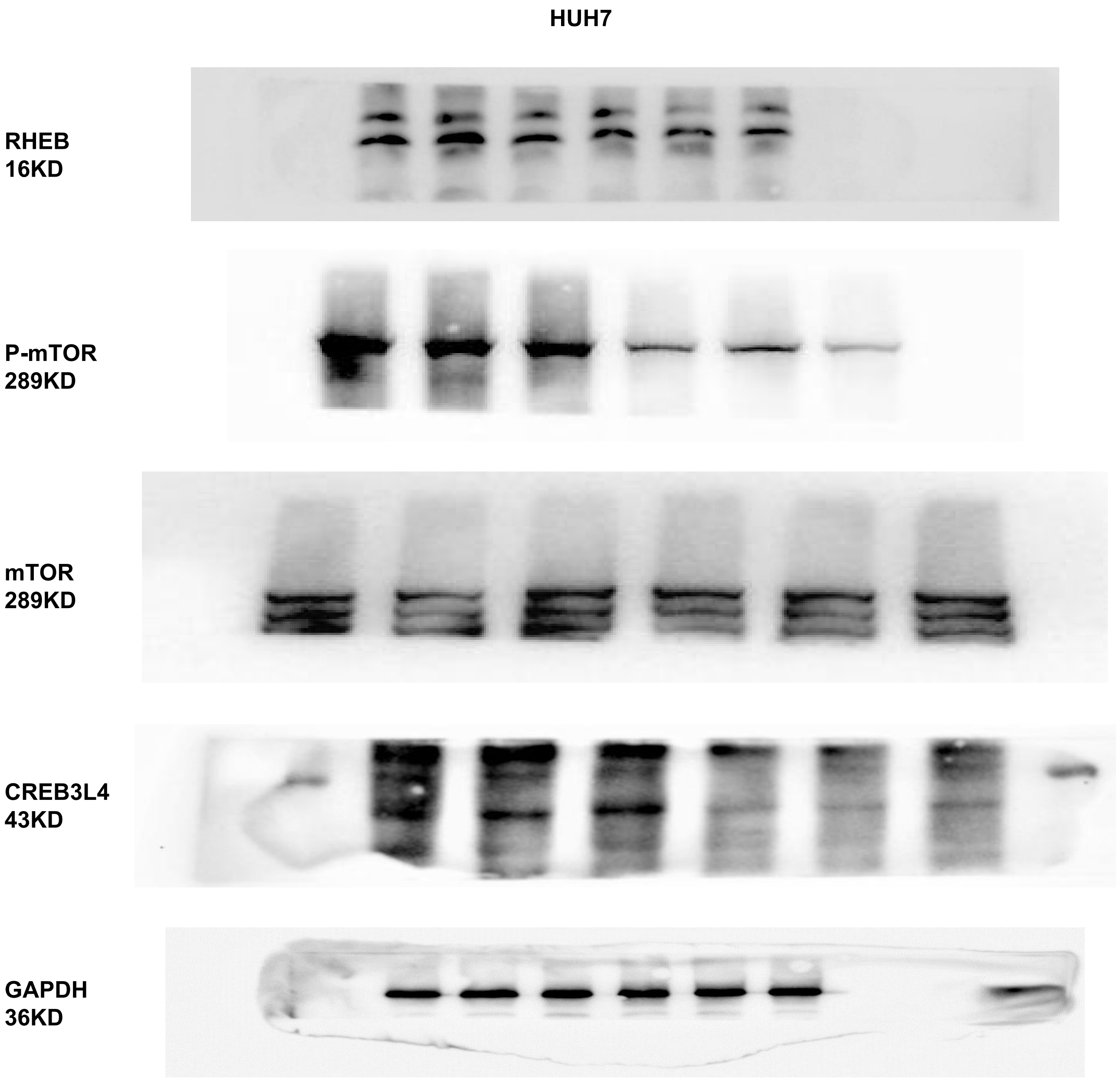

Data S15 Original images of Western blot, related to Figure 6C.

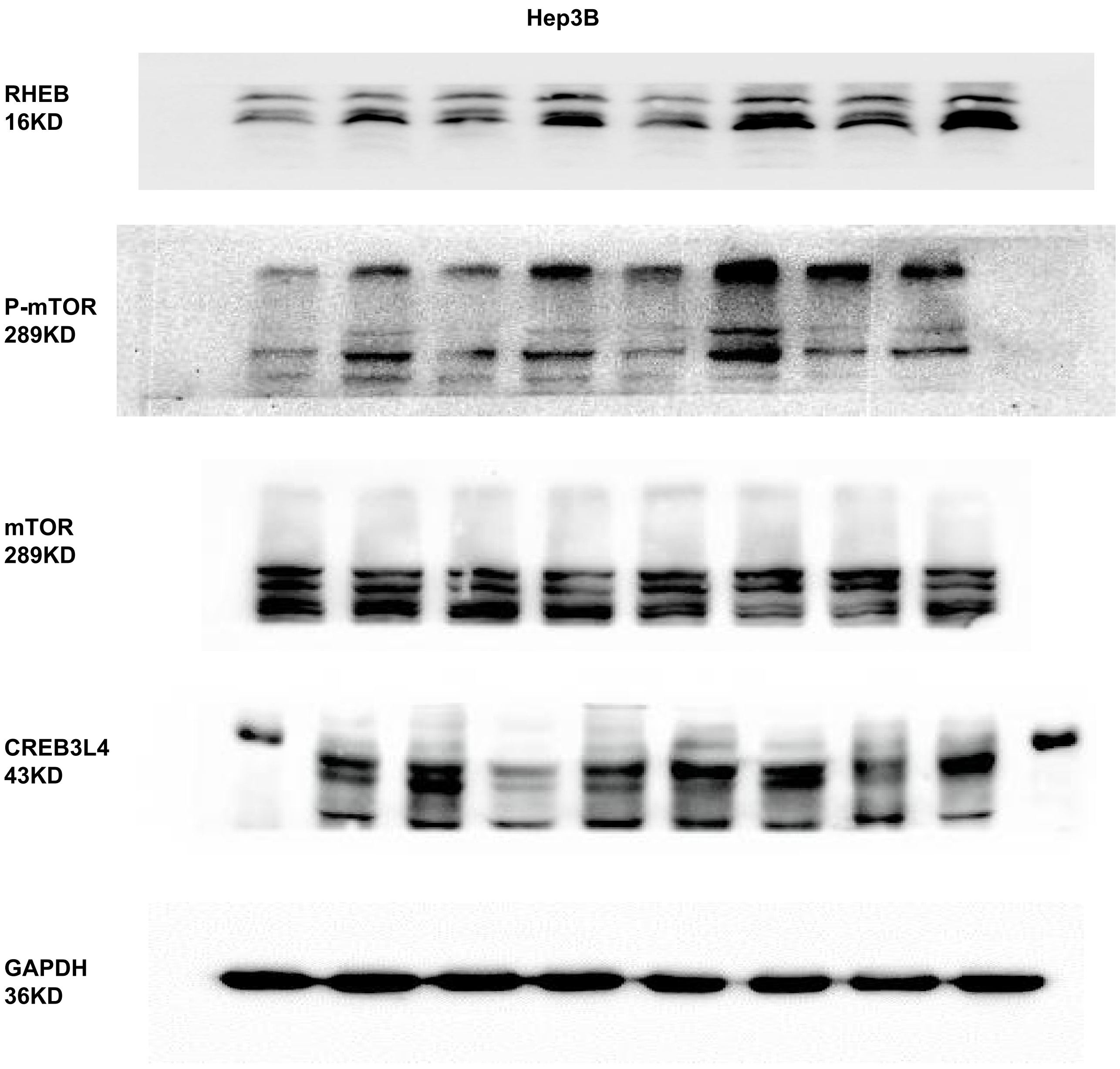

Data S16 Original images of Western blot, related to Figure 6D.

CREB3L4
43KD

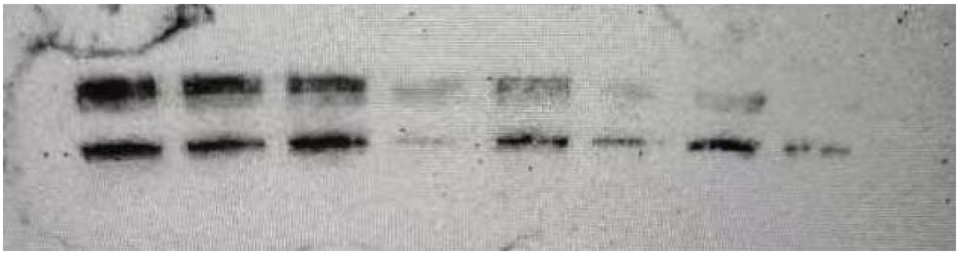

GAPDH
36KD

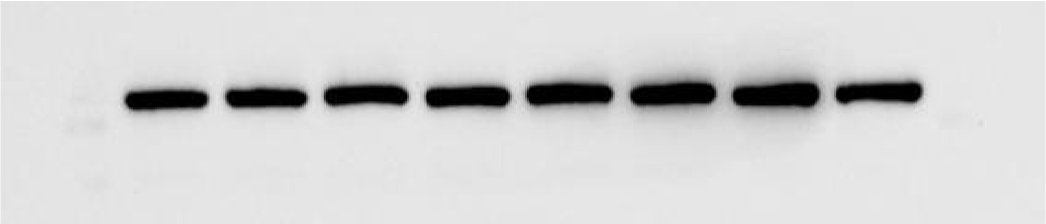

Data S17 Original images of Western blot, related to Supplementary Figure 1A.
